# Construction of Donor–Acceptor Heterojunctions via Microphase Separation of Discotic Liquid Crystals with Ambipolar Transport

**DOI:** 10.3390/molecules30163441

**Published:** 2025-08-21

**Authors:** Heng Liu, Mingsi Xie, Yaohong Liu, Gaojun Jia, Ruijuan Liao, Ao Zhang, Yi Fang, Xiaoli Song, Chunxiu Zhang, Haifeng Yu

**Affiliations:** 1 School of Printing and Packaging Engineering, Beijing Institute of Graphic Communication, Beijing 102600, Chinaxiemingsi@bigc.edu.cn (M.X.); 20230152011@stu.bigc.edu.cn (G.J.); zhangao@bigc.edu.cn (A.Z.); fangyi@bigc.edu.cn (Y.F.);; 2 Key Laboratory of Polymer Chemistry and Physics of Ministry of Education, School of Materials Science and Engineering, Peking University, Beijing 100871, China

**Keywords:** discotic liquid crystal, triphenylene, perylene, donor–acceptor molecule, bulk heterojunction, ambipolar charge carrier transport

## Abstract

A series of novel discotic liquid crystalline donor–acceptor hybrid heterojunctions were prepared by blending the triphenylene derivative (T5E36) as donor and perylene tetracarboxylic esters as acceptor. Mesophases of blends were characterized by using polarized optical microscopy, differential scanning calorimetry, and X-ray diffraction. Results suggest that all the blends formed liquid crystalline phases, where both compounds in the blends self-assembled separately into columns yet cooperatively contributed to the overall hexagonal or tetragonal columnar mesophase structure. The charge carrier mobilities were characterized using a time-of-flight technique. The phase-separated columnar nanostructures of the donor and acceptor components play an important role in the formation of molecular heterojunctions exhibiting highly efficient ambipolar charge transport, with mobilities on the order of 10^−3^ cm^2^ V^−1^ s^−1^. These blends with ambipolar transport properties have great potential for application in non-fullerene organic solar cells, particularly in bulk heterojunction architectures.

## 1. Introduction

Molecular self-assembly into ordered and functional structures [1], a phenomenon in nature, plays an indispensable role across diverse fields such as chemistry, biology, and materials science [2,3,4,5], which is primarily driven by non-covalent interactions, including π–π stacking, van der Waals forces, and hydrogen bonding [6,7,8,9]. This bottom-up approach can construct complex structures through molecular recognition and complementarity [10]. Liquid crystals (LCs) are not only a self-assembly paradigm but also represent a classic type of soft matter [11,12,13]. As molecular structure plays a key role in the self-assembling process, rational molecular design combined with controlled self-assembly enables the development of LC materials with novel functionalities [14,15,16,17], thereby advancing their applications in the field of organic electronics.

In 1977, Chandrasekhar [18] first discovered discotic LCs (DLCs) composed of planar aromatic cores with multiple peripheral flexible chains [19,20]; this structure facilitates strong π–π conjugation between the planar aromatic cores, which promotes the molecules to stack in a direction perpendicular to the core, forming one-dimensional (1D) columnar structures. In 1978, J. Billard [21] successfully synthesized hexa(alkoxy)triphenylenes, establishing triphenylene derivatives as representative molecules in the field of DLC materials. In 1994, D. Haarer reported the exceptional charge carrier mobility of the hexahexyloxytriphenylene in its helical phase, reaching up to 1 × 10^−1^ cm^2^ V^−1^ s^−1^ [22], which is comparable to that of organic single crystal materials. This study not only demonstrates its potential for application in organic electronic devices but also further stimulates considerable interest and extensive research in the development of novel DLCs. The triphenylene core is readily functionalized at its β-positions (2, 3, 6, 7, 10, and 11), which imparts structural tunability and processability. Through π–π stacking, it forms highly ordered 1D columnar assemblies that are electron-rich and exhibit excellent hole-transporting properties. As a result, triphenylene derivatives are commonly used as electron-donating materials.

**Figure 1:**
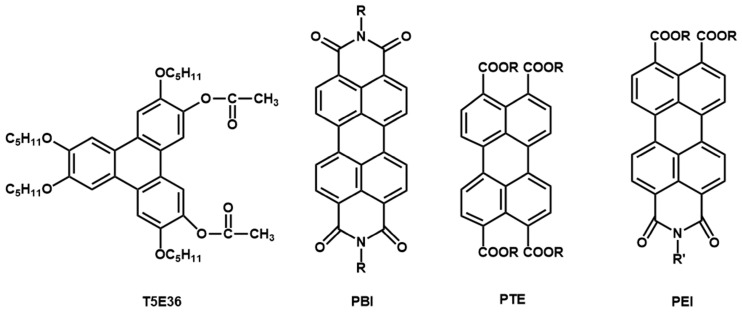
Chemical structures of the compounds: 3,6-di(ethoxycarbonyl)-2,7,10,11-tetrapentyloxytriphenylene (T5E36), perylene bisimide (PBI), perylene tetracarboxylic ester (PTE), and perylene ester imide (PEI).

Perylene and its derivatives are indispensable organic electronic materials [23,24,25,26,27] and have attracted considerable attention due to their low cost, excellent chemical stability, and ease of functionalization. These compounds exhibit several favorable photophysical and electronic properties, including a wide bandgap, high molar extinction coefficient, strong electron affinity, and high fluorescence quantum yield [26,28,29]. Consequently, they have been widely regarded as promising materials for applications in organic solar cells, organic light-emitting diodes, and organic field-effect transistors. Perylene-based LC materials can be broadly classified into three main categories: perylene bisimides (PBIs), perylene tetracarboxylic esters (PTEs), and perylene ester imides (PEIs) (Figure 1). In 1997, Cormier and co-workers [30,31] first reported LC perylene bisimides (PBIs), marking them as both the earliest synthesized and most well-known class of perylene derivatives. PBIs have found significant industrial applications owing to their strong light absorption, excellent thermal, chemical, and photochemical stability [32,33], making them suitable for use as dyes and pigments. Moreover, their high electron affinity and strong fluorescence properties have sparked substantial research interests. However, the application of PBIs in LC materials remains limited due to their extremely low solubility and lack of sufficient flexible side chains. In contrast, the second class of perylene derivatives, PTEs, and the third class, asymmetrical PEIs, exhibit significantly improved solubility. The incorporation of alkyl chains imparts flexibility to the molecular structure, facilitating self-assembly and promoting the formation of columnar LC phases that can remain stable over a wide temperature range, including at room temperature [23,29,34,35]. In particular, PTEs can be synthesized with high yields and purity, and their highly conjugated perylene core, combined with the presence of four electron-withdrawing ester groups, endows them with excellent electron affinity and a low LUMO energy level [36], making them highly attractive as n-type (electron-transporting) semiconductors.

In 1986, perylene tetracarboxylic acid derivatives (PV) and copper phthalocyanine (CuPc) were first reported by C.W. Tang as the photoelectric active material layer of a double-layer organic solar cell, achieving charge generation and separation through a double-layer structure [37], demonstrating the potential of perylene-based materials in organic solar cells at that time. However, due to limitations in the interface region, bilayer organic solar cells exhibit deficiencies in exciton dissociation efficiency. Subsequently, in 1995, A.J. Heeger and his team introduced the concept of an internal donor–acceptor heterojunction network [38]. Compared to the traditional bilayer structure, the bulk heterojunction significantly increased the donor/acceptor (D/A) interface area, thereby promoting efficient photoinduced charge separation and carrier collection efficiency. The innovative design of the bulk heterojunction led to a significant improvement in the efficiency of organic solar cells, becoming the core architecture of organic photovoltaic devices over the past three decades. In 2009, Mikroyannidis proposed a hybrid bulk heterojunction organic solar cell based on phenylenevinylene copolymers, PBIs, and TiO_2_ nanoparticles, achieving a relatively high power conversion efficiency of 2.64% [39]. Later, in 2013, Sheng Qiang Xiao and his colleagues replaced PBI with PTE as the polymer acceptor unit, which significantly improved the open-circuit voltage (Voc reached 0.83 V) [40], providing a new direction for the design of bulk heterojunction solar cells. We previously reported that 3,6-di(ethoxycarbonyl)-2,7,10,11-tetrapentyloxytriphenylene exhibited a broad-temperature-range hexagonal columnar mesophase, maintaining a high degree of order even at room temperature [41,42]. As a hole-transporting material, it displays excellent *p*-type conductivity. In contrast, PTEs serve as electron-transporting materials with strong fluorescence and *n*-type semiconducting properties. Therefore, in this work, we focus on adjusting the LC phase behavior of the blend systems composed of 3,6-di(ethoxycarbonyl)-2,7,10,11-tetrapentyloxytriphenylene and PTEs, as well as their impact on optoelectronic performance. Our study provides new insights for the design of organic solar cells with excellent LC and optoelectronic properties, particularly for applications in bidirectional charge-transporting bulk heterojunction devices.

## 2. Results and Discussion

### 2.1. Synthesis of 3,6-di(Ethoxycarbonyl)-2,7,10,11-Tetrapentyloxytriphenylene and PTE

**Scheme 1:**
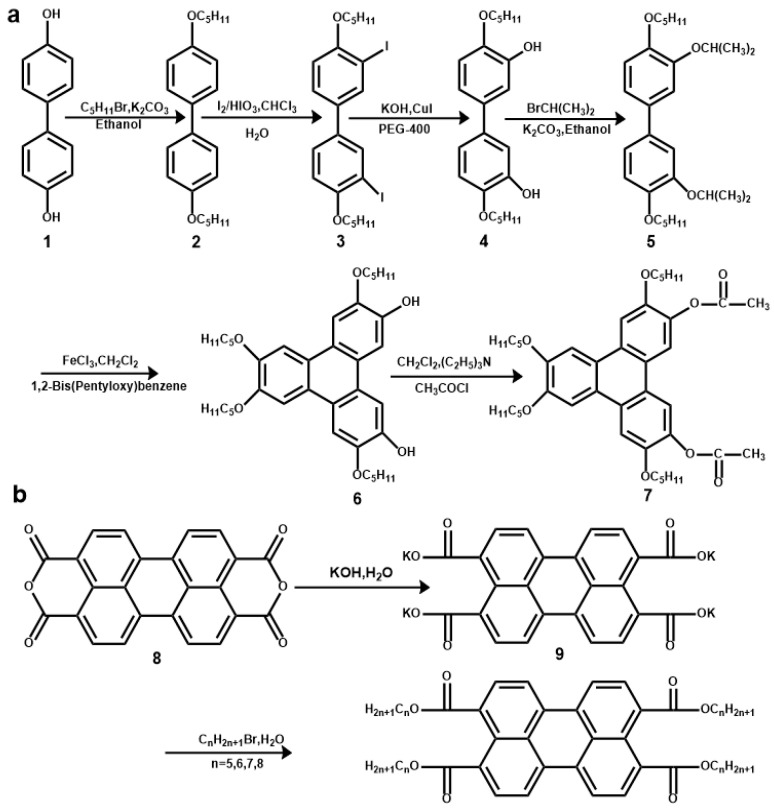
(**a**) Synthetic route of the triphenylene derivative T5E36 and (**b**) synthetic route of PETCn.

The synthesis is described in detail in the Supplementary Materials. The molecular characterization data of the compounds are provided in the Supplementary Materials (Figures S1–S5 and S8). In brief, the synthesis of the triphenylene derivative, 3,6-di(ethoxycarbonyl)-2,7,10,11-tetrapentyloxytriphenylene, is shown in Scheme 1a. This compound is abbreviated as T5E36, where “T” refers to the triphenylene core, “5” to the pentyloxy side chains, “E” to the ester groups, and “3,6” to the substitution positions of the ester functionalities. The synthetic route was adapted from our group’s previous work reported in 2014, in which 3,6-dihydroxy-2,7,10,11-tetrapentyloxytriphenylene was synthesized with a yield of up to 24.7% [43], providing a key intermediate for the current study. After obtaining the dihydroxy compound, it was reacted under a nitrogen atmosphere in anhydrous dichloromethane with triethylamine and ethyl chloroformate at 40 °C under reflux for 24 h. The reaction mixture was subsequently extracted, purified by column chromatography, and recrystallized from ethanol to afford the desired white product in a yield of 95%.

The synthetic route for 3,4,9,10-tetracarboxylic acid tetraalkyl ester of perylene (PTECn) is shown in Scheme 1b, where n denotes the number of carbon atoms in the ester side chains. Specifically, *n* = 5, 6, 7, and 8 correspond to 3,4,9,10-tetrakis (n-pentyl ester) perylene tetracarboxylate, 3,4,9,10-tetrakis (n-hexyl ester), 3,4,9,10-tetrakis (n-heptyl ester), and 3,4,9,10-tetrakis (n-octyl ester) perylene tetracarboxylates, respectively.

Synthesis of perylene-3,4,9,10-tetracarboxylic acid potassium salt is as follows. In a 250 mL three-neck flask, perylene-3,4,9,10-tetracarboxylic dianhydride was added to deionized water and stirred magnetically for 10 min. Potassium hydroxide was then slowly added to the resulting suspension, and the mixture was heated to reflux for 4 h until the red solid completely dissolved. After cooling to room temperature, the reaction mixture was slowly poured into methanol. The resulting yellow solid was collected by vacuum filtration using a Büchner funnel, affording the product in a yield of 99%.

Synthesis of 3,4,9,10-tetrakis (n-pentyl ester) perylene tetracarboxylate (PTEC5) is presented below as a representative example. In a three-neck round-bottom flask, perylene-3,4,9,10-tetracarboxylic acid potassium salt, potassium carbonate, methyltrioctylammonium chloride, potassium iodide, and deionized water were added in sequence. The mixture was stirred magnetically for 10 min, after which n-pentyl bromide was added dropwise via a pressure-equalizing dropping funnel. The reaction mixture was refluxed for 24 h and then cooled to room temperature. The product was extracted three times with dichloromethane, and the combined organic layers were dried over anhydrous sodium sulfate overnight. After removing the solvent under reduced pressure, the crude product was purified by column chromatography, followed by recrystallization from ethanol to afford a golden-yellow solid in 89% yield. PTEC6, PTEC7, and PTEC8 were synthesized following a similar procedure to that described for PTEC5.

In this study, four binary blend systems—T5E36/PTEC5, T5E36/PTEC6, T5E36/PTEC7, and T5E36/PTEC8—were prepared by mixing 3,6-di(ethoxycarbonyl)-2,7,10,11-tetrapentyloxytriphenylene (T5E36) with PTEC5, PTEC6, PTEC7, and PTEC8, respectively, in a 1:1 molar ratio. T5E36 (0.2 g, 0.29 mmol) was respectively blended with PTEC5 (0.189 g, 0.29 mmol), PTEC6 (0.205 g, 0.29 mmol), PTEC7 (0.221 g, 0.29 mmol), and PTEC8 (0.238 g, 0.29 mmol) in 20 mL of DCM. Each mixture was stirred for 2 h using a magnetic stirrer. After partial evaporation of DCM at room temperature, the residual solvent was removed in a vacuum drying oven.

### 2.2. Thermal Properties and Phase Behaviors of T5E36 and PTECn

**Figure 2:**
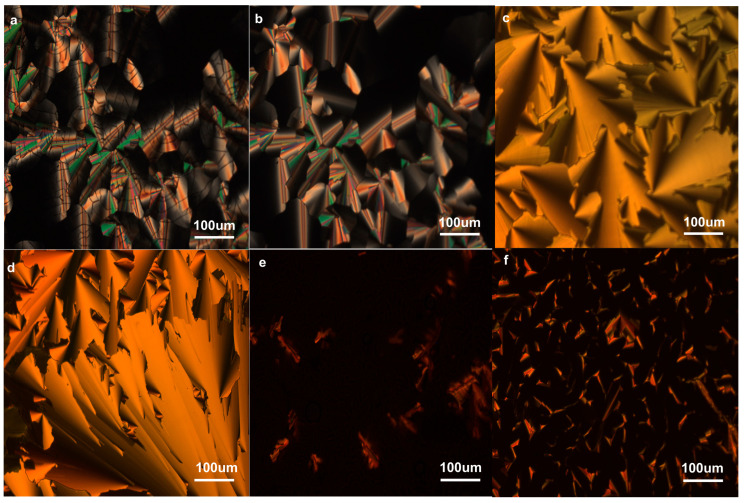
POM images of the components: (**a**) T5E36 at 40 °C, (**b**) T5E36 at 125 °C, (**c**) PTEC5 at 90 °C, (**d**) PTEC6 at 90 °C, (**e**) PTEC7 at 90 °C, and (**f**) PTEC8 at 110 °C.

The LC phase behavior of T5E36 and PTECn (*n* = 5, 6, 7, 8) was analyzed and characterized using polarized optical microscopy (POM) and differential scanning calorimetry (DSC). Variable-temperature wide-angle X-ray diffraction (WAXD) was employed to characterize the structural organization of the samples. Upon cooling at a rate of 10 °C/min, T5E36 exhibited a focal conic fan texture at 125 °C, which is indicative of a typical hexagonal columnar (Col_h_) phase, and this texture remained stable down to room temperature. PTEC5, PTEC6, PTEC7, and PTEC8 all displayed fan-shaped textures in the high-temperature LC region under POM, see Figure 2, also characteristic of the Col_h_ phase. Upon further cooling to room temperature, these materials transitioned into crystalline states.

**Figure 3:**
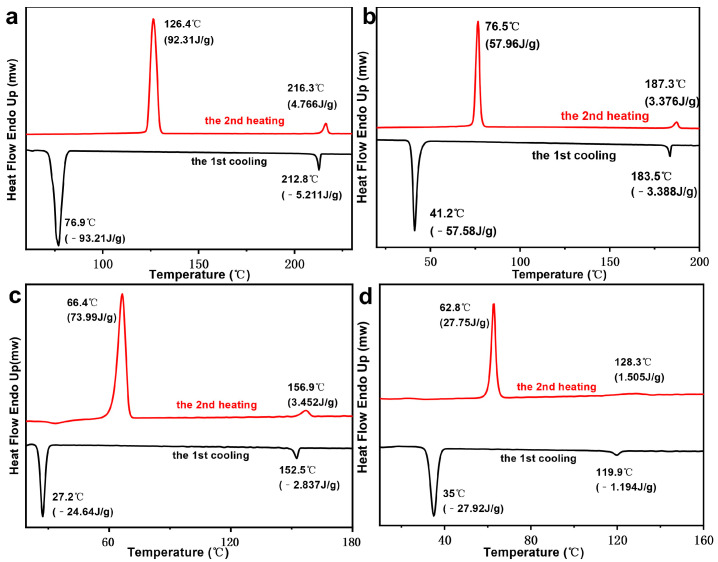
DSC thermograms of (**a**) PTEC5, (**b**) PTEC6, (**c**) PTEC7, and (**d**) PTEC8 recorded during heating and cooling cycles at a rate of 10 °C/min.

T5E36 exhibited two endothermic peaks during the heating process at 135 °C (ΔH = 6.659 J/g) and 172 °C (ΔH = 26.39 J/g) and two exothermic peaks at 167.5 °C (ΔH = −25.65 J/g) and 123 °C (ΔH = −4.535 J/g) during the first cooling process (Figure S11). Taking PTEC7 as an example, two endothermic peaks were observed at 66.4 °C (ΔH = 73.99 J/g) and 156.9 °C (ΔH = 3.452 J/g) during the heating process. During the first cooling process, two exothermic peaks were detected: the first at 152.5 °C (ΔH = −2.837 J/g), corresponding to the transition from the isotropic phase to the hexagonal columnar Col_h_ phase, and the second at 27.2 °C (ΔH = −24.64 J/g), corresponding to the transition from the Col_h_ phase to the crystalline state (Figure S12); PTEC5, PTEC6, and PTEC8 exhibited similar DSC thermal behaviors (Figure 3).

**Figure 4:**
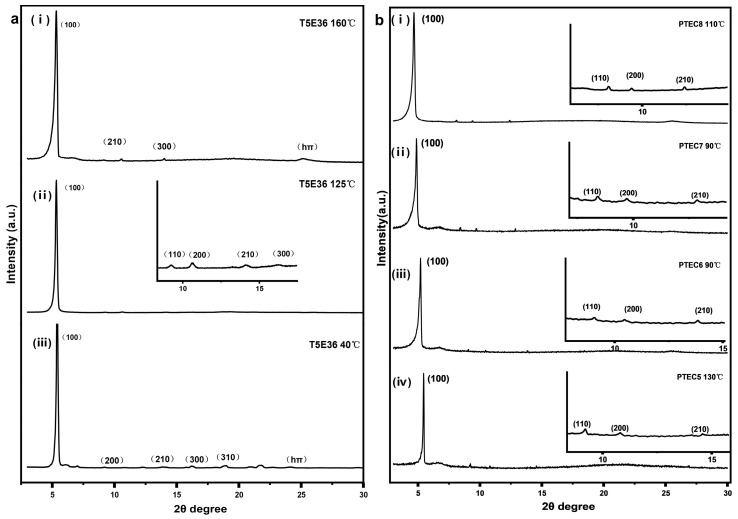
(**a**) WAXD patterns of T5E36 at different temperatures: (**i**) 160 °C, (**ii**) 125 °C, and (**iii**) 40 °C; (**b**) WAXD patterns of PTECn at different temperatures: (**i**) PTEC8 at 110 °C, (**ii**) PTEC7 at 90 °C, (**iii**) PTEC6 at 90 °C, and (**iv**) PTEC5 at 130 °C.

The intermediate phases of T5E36, PTEC5, PTEC6, PTEC7, and PTEC8 were characterized by WAXD (Figure 4). Diffraction patterns were recorded at various temperatures during cooling at a rate of 10 °C/min. For example, the WAXD patterns of T5E36 obtained at 160 °C, 125 °C, and 40 °C exhibited sharp diffraction peaks in the 2θ = 5–20° range, which could be indexed as the (100), (110), (200), and (210) reflections with a d-spacing ratio of 1:√3:√4:√7, confirming a hexagonal packing structure. Taking PTEC7 as a representative example, a strong diffraction peak was observed in the small-angle region at 2θ = 4.85°, corresponding to the (100) reflection with a calculated d-spacing of 18.20 nm. In the range of 2θ = 5–20°, four diffraction peaks were observed at d = 18.20 nm, 10.44 nm, 9.16 nm, and 6.88 nm. These peaks exhibit a spacing ratio of 1:√3:√4:√7 and can be indexed as the (100), (110), (200), and (210) reflections, confirming the presence of a Col_h_ phase due to hexagonal molecular packing. PTEC5, PTEC6, and PTEC8 displayed similar WAXD patterns to that of PTE-C7, indicating that they also adopt the Col_h_ phase in the high-temperature region.

### 2.3. Mesophases of T5E36/PTECn Blend Systems

The LC phase behavior of T5E36/PTECn (*n* = 5, 6, 7, 8) was investigated and characterized by POM and DSC. Variable-temperature WAXD was employed to analyze the structural organization of the resulting materials.

**Figure 5:**
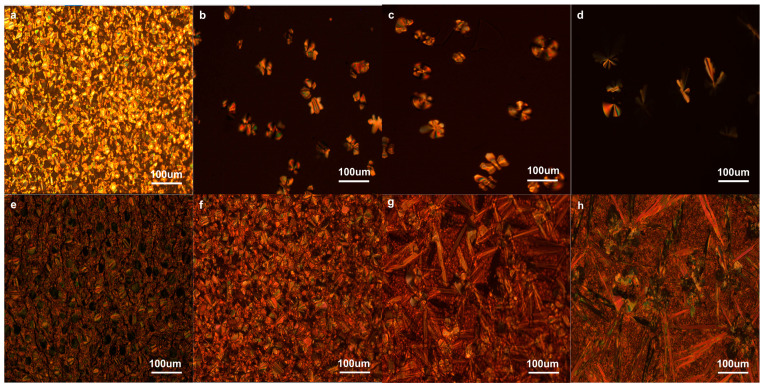
POM images of T5E36/PTEC5, T5E36/PTEC6, T5E36/PTEC7, and T5E36/PTEC8 upon cooling from the isotropic phase: (**a**) T5E36/PTEC5 at 130 °C; (**b**) T5E36/PTEC6 at 120 °C; (**c**) T5E36/PTEC7 at 120 °C; (**d**) T5E36/PTEC8 at 120 °C; (**e**) T5E36/PTEC5 at 40 °C; (**f**) T5E36/PTEC6 at 80 °C; (**g**) T5E36/PTEC7 at 80 °C; (**h**) T5E36/PTEC8 at 40 °C.

The phase behaviors of the four blend systems were characterized by POM (Figure 5). Upon cooling at a rate of 10 °C/min from the high-temperature LC region, T5E36/PTEC6, T5E36/PTEC7, and T5E36/PTEC8 exhibited typical focal conic textures, indicative of a Col_h_ phase; T5E36/PTEC5 displayed a broken fan-shaped texture, characteristic of a rectangular columnar (Col_r_) phase. Upon further cooling, the textures of T5E36/PTEC6 and T5E36/PTEC7 transformed into broken fan-shaped patterns, indicative of a Col_r_ phase, while T5E36/PTEC5 and T5E36/PTEC8 transitioned into a crystalline state.

**Figure 6:**
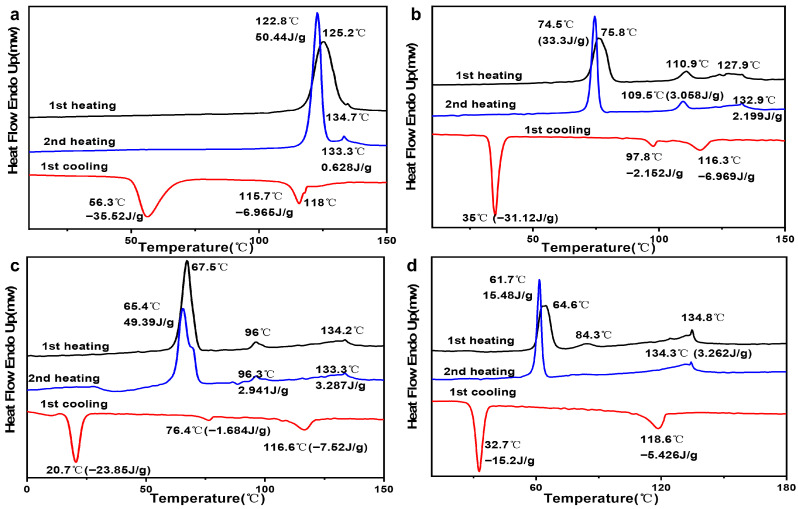
DSC thermograms of (**a**) T5E36/PTEC5, (**b**) T5E36/PTEC6, (**c**) T5E36/PTEC7, and (**d**) T5E36/PTEC8 recorded during cooling and heating cycles at a rate of 10 °C/min.

The thermal dynamic behaviors of the four blend systems were characterized by the DSC method (Figure 6). Taking T5E36/PTEC7 as a representative example, during the first cooling process, three exothermic peaks were observed at 116.6 °C (ΔH = −7.52 J/g), 76.4 °C (ΔH = −1.684 J/g), and 20.7 °C (ΔH = −23.85 J/g). Three thermal transitions were observed during the second heating scan, as shown in Figure 6c. The peak at 65.4 °C (ΔH = 49.39 J/g) corresponds to the transition from the crystalline state to the Col_r_ phase. The peak at 96.3 °C (ΔH = 2.941 J/g) is attributed to the transition from the Col_r_ phase to the Col_h_ phase, while the peak at 133.35 °C (ΔH = 3.287 J/g) corresponds to the transition from the Col_h_ phase to the isotropic phase. T5E36/PTEC6 exhibits a similar thermal behavior. For T5E36/PTEC8, two peaks were observed during the second heating scan. The first peak at 61.7 °C (ΔH = 15.45 J/g) corresponds to the transition from the crystalline state to the Col_h_ phase, and the second peak at 134.3 °C (ΔH = 3.262 J/g) corresponds to the transition from the Col_h_ phase to the isotropic phase. For T5E36/PTEC5, two thermal transitions were observed during the second heating scan. The first peak, at 122.8 °C (ΔH = 50.44 J/g), corresponds to the transition from the crystalline state to the Col_r_ phase. The second peak, at 133.3 °C (ΔH = 0.628 J/g), is attributed to the transition from the Col_r_ phase to the isotropic phase.

The first and second heating curves of all four blend systems show good overlap, indicating that the blends form stable systems. This suggests that no microphase separation occurred between T5E36 and PTECn and that the two components are well-mixed and fully miscible. These results confirm that all four blends can form a homogeneous heterojunction structure.

**Figure 7:**
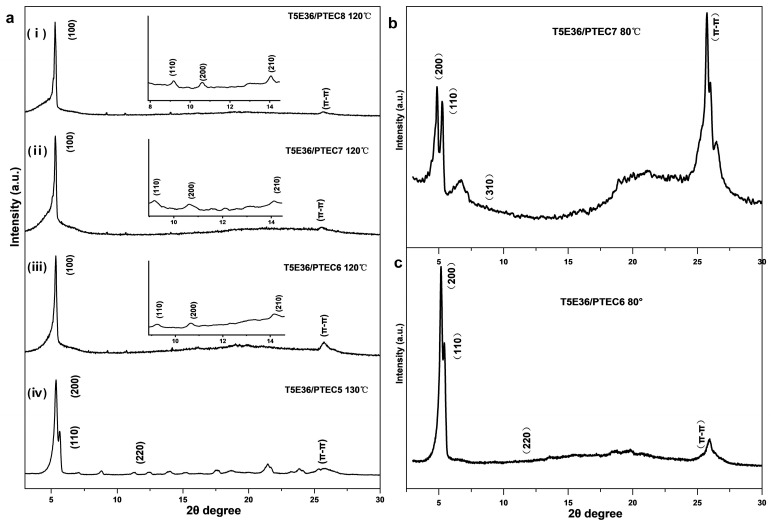
(**a**) WAXD patterns of T5E36/PTECn: (**i**) T5E36/PTEC8 at 120 °C, (**ii**) T5E36/PTEC7 at 120 °C, (**iii**) T5E36/PTEC6 at 120 °C, and (**iv**) T5E36/PTEC5 at 130 °C; (**b**) WAXD patterns of T5E36/PTEC7 at 80 °C; (**c**) WAXD patterns of T5E36/PTEC6 at 80 °C.

The intermediate phases of the blends were characterized by WAXD (Figure 7). Diffraction patterns were recorded at various temperatures during cooling at a rate of 10 °C/min. Taking T5E36/PTEC7 as a representative example, at 120 °C, four distinct diffraction peaks were observed in the small-angle region (2θ = 5–20°), corresponding to d-spacings of 16.62 nm, 9.60 nm, 8.28 nm, and 6.28 nm. The ratios of the d-spacings follow 1:√3:√4:√7, which can be indexed as the (100), (110), (200), and (210) reflections, respectively. These results confirm the presence of a Col_h_ phase attributed to hexagonal packing. In the wide-angle region, a broad peak at 2θ = 21.07° and a sharp peak at 25.46° were observed, which are attributed to the π–π stacking of the aromatic cores, respectively. As shown in Table 1, the lattice parameter a was calculated from the d_100_ spacing using the following Equation (1):

1/d^2^_hk_ = 4 (h^2^ + k^2^ + hk)/3a^2^ (1)

T5E36/PTEC6 and T5E36/PTEC8 exhibited similar WAXD patterns to that of T5E36/PTEC7, as shown in Figure 7a(i,iii), indicating that they can also be considered Col_h_ phase in the high temperature range. At 130 °C, T5E36/PTEC5 exhibited two sharp and well-separated peaks in the small-angle region (2θ = 5°–6°), corresponding to the splitting of the (100) reflection into (200) and (110) reflections; the diffraction peak at 2θ = 11.31° (d = 7.81) is indexed as the (220) reflection. In the wide-angle region, the peak around 2θ ≈ 25° corresponds to the (001) reflection, which is associated with the π–π stacking of the discotic cores. This pattern is characteristic of a Col_r_ phase [44]. The lattice constants a and b were calculated based on the d_110_ and d_200_ values using Equation (2).

1/d^2^_hkl_ = h^2^/a^2^ + k^2^/b^2^ (2)

For the Col_r_ model, two possible two-dimensional (2D) plane group symmetries can be considered as *c*2*mm* and *p*2*gg*. For the *c*2*mm* symmetry, all (*hk*) reflections must additionally satisfy the condition *h* + *k* = 2*n*; for the *p*2*gg* symmetry, all (*hk*) reflections must additionally satisfy the condition *h* + *k* = 2*n +* 1. Based on the analysis of the WAXD patterns, we identified the structure as belonging to the *c*2*mm* 2D plane group. Upon further cooling to 80 °C at the same rate, T5E36/PTEC6 exhibited two sharp and well-separated peaks in the small-angle region (2θ = 5–6°), corresponding to the splitting of the (100) reflection into the (200) and (110) reflections. A peak at 2θ = 10.9° (d = 8.11 Å) can be indexed as the (200) reflection. This diffraction pattern is characteristic of a Col_r_ phase with a *c*2*mm* symmetry. T5E36/PTEC7 displayed a similar WAXD pattern at 80 °C.

**Figure 8:**
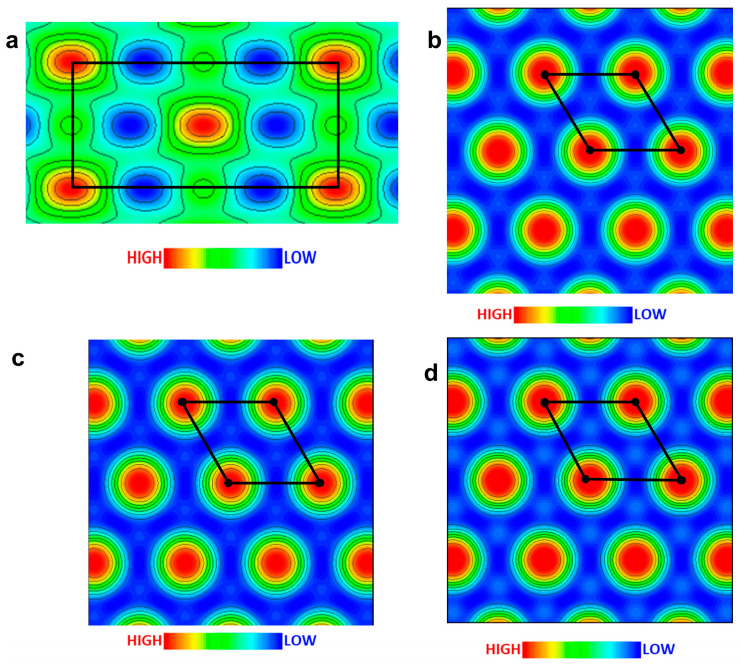
Reconstructed electron density maps of (**a**) T5E36/PTEC5, (**b**) T5E36/PTEC6, (**c**) T5E36/PTEC7, and (**d**) T5E36/PTEC8 with a color scale from high density (red) to low density (blue).

To investigate the model of T5E36/PTECn blends deeply, the electron density map (EDM) is reconstructed from XRD data, see Figure 8. The electron density map of T5E36/PTEC5 corresponds to a rectangular columnar phase with *c*2*mm* symmetry; T5E36/PTEC6, T5E36/PTEC7, and T5E36/PTEC8 exhibit hexagonal columnar (Col_h_) phases. The red area with the highest electron density represents the location of the aromatic core, while the surrounding yellow, green, and blue areas are filled with ester groups and alkyl tails. In the electron density map of the *c*2*mm* rectangular columnar phase, the cross-section of the columnar core (red region) appears more elliptical compared to the circular one observed in the hexagonal columnar phase. The rectangular lattice units are elongated along the a-axis and slightly compressed along the b-axis [45,46].

### 2.4. Steady-State Spectrum

**Figure 9:**
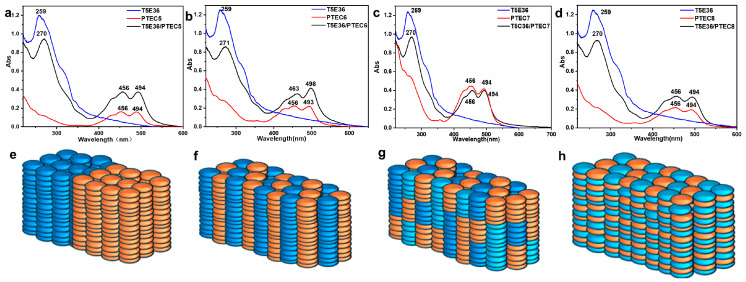
UV–vis absorption spectra of (**a**) T5E36, PTEC5, T5E36/PTEC5, (**b**) T5E36, PTEC6, T5E36/PTEC6, (**c**) T5E36, PTEC7, T5E36/PTEC7, and (**d**) T5E36, PTEC8, T5E36/PTEC8 in DCM at a concentration of 1.1 × 10^−5^ M. Panels (**e**–**h**) illustrate schematic representations of four types of nanoscale phase separation: (**e**) macroscopic phase separation, (**f**) intercolumnar separation, (**g**) intracolumnar separation, and (**h**) alternating donor–acceptor stacking within a single column.

UV–vis absorption spectra were measured for T5E36, PTEC5, PTEC6, PTEC7, PTEC8, and their corresponding blends (T5E36/PTEC5, T5E36/PTEC6, T5E36/PTEC7, T5E36/PTEC8) in dichloromethane (DCM) at a dilution concentration of 1.1 × 10^−5^ M (Figure 9a–d).

Taking T5E36/PTEC7 as an example (Figure 9c), T5E36 exhibits absorption in the range of 240–320 nm, with a maximum peak at 259 nm. PTEC7 shows a broad absorption band in the range of 410–510 nm. In the blended T5E36/PTEC7 system, the maximum absorption at 270 nm is attributed to the π–π* transition and exhibits a slight red shift. The absorption band between 400 and 600 nm corresponds to characteristic transitions of the perylene core. Specifically, the maxima at 456 nm and 494 nm are consistent with those of PTEC7, corresponding to the S0 → S1 (0,0) and (0,1) vibronic transitions, respectively. When T5E36 and PTECn are co-assembled within the same columnar stack, electrostatic D-A interactions may induce the formation of charge transfer (CT) complexes, which would result in a new CT absorption band. However, in the UV–vis absorption spectrum of T5E36/PTEC7, neither distinct changes in the absorption maximum nor the emergence of a CT band were observed in the ground state. The spectrum appears to be a simple superposition of the individual spectra of the donor and acceptor, indicating that T5E36 and PTEC7 are stacked in separate columns rather than in a mixed D-A column. This suggests nanoscale phase separation between the two components. Similar absorption behavior was observed for T5E36/PTEC5, T5E36/PTEC6, and T5E36/PTEC8, confirming that T5E36 and PTECn are self-assembled into separate columnar domains, as shown in Figure 9f.

To further verify the type of interaction between T5E36 and PTECn in the blending process, FT-IR measurements were carried out on the individual components as well as the blended films, as shown in Figure S13. The absorption peaks of the blend closely match those of the individual components, with no new characteristic peaks or significant peak shifts observed. This suggests that no charge-transfer complex was formed during the blending process. The fluorescence emission spectrum after mixing has the emission characteristics of T5E36 and PTECn. Taking T5E36/PTEC7 as an example, as shown in Figure S14c, its fluorescence shows a maximum peak corresponding to T5E36 at 380 nm and a maximum peak corresponding to PTEC7 at 520 nm. Before and after mixing, the fluorescence spectrum does not show a red shift, so there is no energy transfer between T5E36 and PTEC7 molecules, which also further confirms that T5E36 and PTECn are stacked in two independent columns. Other T5E36/PTECn have similar fluorescence spectra (Figure S14).

### 2.5. Charge Carrier Mobility

The electron and hole mobilities of T5E36/PTEC5, T5E36/PTEC6, T5E36/PTEC7, and T5E36/PTEC8 were measured using the time-of-flight (TOF) method, confirming the ambipolar transport properties of the T5E36/PTECn systems. For measurements, the samples were first heated above their clearing temperatures and then introduced in the liquid state into an LC cell composed of two ITO-coated glass plates with a gap of 9 μm via capillary action. After filling, the cell was cooled to room temperature at a rate of 0.5 °C/min. A pulsed laser with a wavelength of 337 nm was used to excite the sample, and the resulting photocurrent was amplified and monitored using a digital oscilloscope. The carrier transit time was determined from the inflection point of the transient photocurrent curve plotted on a double logarithmic scale. The charge carrier mobility was calculated using Equation (3):

μ = d^2^/Vt (3)

**Figure 10:**
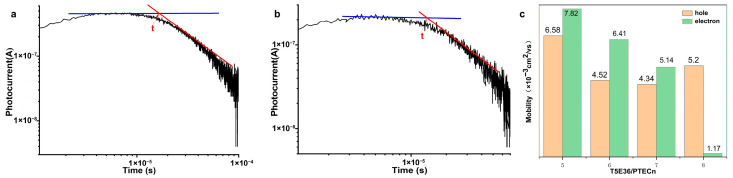
Double logarithmic plots of transient current (I) versus time (t) for T5E36/PTEC5 at 20 °C under an applied electric field of E = 2 × 10^4^ V/cm: (**a**) hole mobility of T5E36/PTEC5; (**b**) electron mobility of T5E36/PTEC5. (**c**) Bar chart of hole and electron mobilities for T5E36/PTEC5, T5E36/PTEC6, T5E36/PTEC7, and T5E36/PTEC8.

In the mobility calculations, V represents the applied voltage (30 V), and d denotes the thickness of the LC cell. As shown in Figure 10a,b, the hole and electron mobilities of T5E36/PTEC5 are 6.58 × 10^−3^ cm^2^ V^−1^ s^−1^ and 7.82 × 10^−3^ cm^2^ V^−1^ s^−1^, respectively. For T5E36/PTEC6, the hole and electron mobilities are 4.52 × 10^−3^ cm^2^ V^−1^ s^−1^ and 6.41 × 10^−3^ cm^2^ V^−1^ s^−1^; for T5E36/PTEC7, the values are 4.34 × 10^−3^ cm^2^ V^−1^ s^−1^ and 5.14 × 10^−3^ cm^2^ V^−1^ s^−1^; and for T5E36/PTEC8, the values are 5.20 × 10^−3^ cm^2^ V^−1^ s^−1^ and 1.17 × 10^−3^ cm^2^ V^−1^ s^−1^, respectively (Figure S15). The carrier mobilities of the T5E36/PTECn systems are approximately one order of magnitude higher than those reported for typical D-A systems. This enhancement is attributed to the well-organized, phase-separated columnar nanostructures of donor and acceptor domains, which significantly promote charge transport. Moreover, the hole and electron mobilities confirm the ambipolar transport behavior, which offers a new pathway for the development of bulk heterojunction optoelectronic devices.

### 2.6. Energy Level Measurements

We determined the optical bandgap E_g_ by analyzing the absorption edge wavelength from the UV–vis absorption spectra (Table S2). The ionization potential (IP) of the semiconductor materials was measured using an ionization potential analyzer, which allowed us to calculate the HOMO energy level. By using the obtained HOMO values with the optical bandgap, the LUMO energy level was estimated using Equation (4):

E_LUMO_ = E_HOMO_ + E_g_ (4)

**Figure 11:**
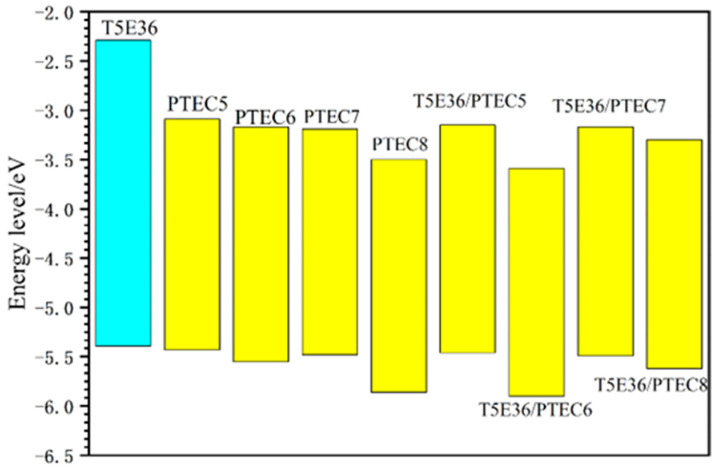
The energy levels for the donor, acceptors, and their blends. The lower end of each column represents the HOMO_UV_ energy level, while the upper end corresponds to the LUMO_UV_ energy level.

The LUMO energy level was calculated accordingly. To gain insight into the HOMO/LUMO energy levels of compounds T5E36 and PTECn, density functional theory (DFT) calculations were carried out using Gaussian 09 (Revision C.01) at the B3LYP/6-31G(d,p) level. The frontier molecular orbitals (HOMO and LUMO) of T5E36 and PTECn are presented in Figures S16–S20. The experimentally determined and calculated energy levels are summarized in Table 2. A schematic diagram of the energy level alignment for the donor (T5E36), the acceptors (PTECn), and their corresponding blends is shown below (Figure 11). The HOMO_UV_ level of the blend lies between that of T5E36 and the corresponding acceptor molecule, but it is closer to the HOMO_UV_ level of T5E36. The LUMO_UV_ level of the blend lies between that of T5E36 and the acceptor, while being closer to that of the acceptor.

## 3. Conclusions

In summary, a series of well-ordered ambipolar molecular D-A bulk heterojunctions, T5E36/PTECn (*n* = 5, 6, 7, 8), were constructed by blending the donor, T5E36, with the acceptor, perylene tetraesters (PTECn), at a 1:1 molar ratio. All the resulting blend systems self-assembled into columnar mesophases, including Col_h_ and Col_r_ phases. UV–vis absorption and fluorescence emission spectra revealed that these D-A blends form nanoscale phase-separated columnar structures through the formation of independent perylene and triphenylene core columns. TOF measurements demonstrated that these blends exhibit relatively high hole and electron mobilities. The D-A systems show clear ambipolar charge transport characteristics, indicating that such ambipolar transport structures can also be achieved through physical blending. Compared with previously reported D-A systems in which ambipolar transport was realized via covalent linkage, this approach simplifies the process of constructing ambipolar transport D-A materials and offers a new strategy for the design of bulk heterojunction optoelectronic devices.

## Figures and Tables

**Table 1:** The summary and detailed indexation of the complementary WAXD data for T5E36/PTEC5, T5E36/PTEC6, T5E36/PTEC7, and T5E36/PTEC8.

Sample	d-Spacing (Å)	Miller Index (*hkl*)	Phase (Lattice Constants)
T5E36/PTEC5 (130 °C)	16.32	(200)	Col_r_ (a = 32.64 Å b = 16.32 Å)
Col_r_/*c*2*mm*	15.71	(110)	
	7.81	(220)	
	3.55	π-π	
T5E36/PTEC6 (120 °C)	16.51	(100)	Col_h_ (a = 19.06 Å)
Col_h_/*p*6*mm*	9.5	(110)	
	8.32	(200)	
	6.21	(210)	
T5E36/PTEC7 (120 °C)	16.62	(100)	Col_h_ (a = 19.19 Å)
Col_h_/*p*6*mm*	9.6	(110)	
	8.28	(200)	
	6.28	(210)	
T5E36/PTEC8 (120 °C)	16.67	(100)	Col_h_ (a = 19.25 Å)
Col_h_/*p*6*mm*	9.62	(110)	
	8.4	(200)	
	6.27	(210)	
T5E36/PTEC6 (80 °C)	17.04	(200)	Col_r_ (a = 34.08 Å b = 18.56 Å)
Col_r_/*c*2*mm*	16.11	(110)	
	8.11	(220)	
	3.45	π-π	
T5E36/PTEC7 (80 °C)	18.16	(200)	Col_r_ (a = 36.32 Å b = 18.83 Å)
Col_r_/*c*2*mm*	16.7	(110)	
	8.7	(310)	
	3.44	π-π	

**Table 2:** Ionization potential (IP), optical bandgap E_g_, HOMO and LUMO energy levels of the compounds and the blend systems. HOMO_UV_ and LUMO_UV_ obtained from the UV–vis absorption spectrum. HOMO_DFT_ and LUMO_DFT_ obtained from gaussian09.

Compound/Blend System	IP (eV)	E_g_ (eV)	HOMO_UV_ (eV)	LUMO_UV_ (eV)	HOMO_DFT_ (eV)	LUMO_DFT_ (eV)
T5E36	5.39	3.1	−5.39	−2.29	−5.177	−0.745
PTEC5	5.55	2.38	−5.55	−3.17	−5.475	−2.670
PTEC6	5.86	2.36	−5.86	−3.05	−5.475	−2.670
PTEC7	5.9	2.31	−5.9	−3.59	−5.473	−2.669
PTEC8	5.62	2.32	−5.62	−3.3	−5.469	−2.665
T5E36/PTEC5	5.43	2.34	−5.43	−3.09		
T5E36/PTEC6	5.48	2.29	−5.48	−3.19		
T5E36/PTEC7	5.46	2.31	−5.46	−3.15		
T5E36/PTEC8	5.49	2.32	−5.49	−3.17		

## Data Availability

The original contributions presented in this study are included in the article/Supplementary Materials. Further inquiries can be directed to the corresponding authors.

## Supplementary Materials

The following supporting information can be downloaded at https://www.mdpi.com/article/10.3390/molecules30163441/s1, Section S1: Materials and Methods; Section S2: Synthesis and Characterization; Section S3: Reconstruction of Relative Electron Density Map; Figure S1: ^1^H-NMR spectrum of T5E36; Figure S2: ^1^H-NMR spectrum of PTEC5; Figure S3: ^1^H-NMR spectrum of PTEC6; Figure S4: ^1^H-NMR spectrum of PTEC7; Figure S5: ^1^H-NMR spectrum of PTEC8; Figure S6: ^13^C-NMR spectrum of T5E36; Figure S7: ^13^C-NMR spectrum of PTEC5; Figure S8: ^13^C-NMR spectrum of PTEC6; Figure S9: ^13^C-NMR spectrum of PTEC7; Figure S10: ^13^C-NMR spectrum of PTEC8. Figure S11: DSC thermogram of T5E36 recorded during heating and cooling cycles at a rate of 10 °C/min; Figure S12: WAXD patterns of PTEC5 at 30 °C, PTEC6 at 30 °C, PTEC7 at 25 °C and PTEC8 at 30 °C; Table S1: Summary and detailed indexation of the complementary WAXD data for T5E36, PTEC5, PTEC6, PTEC7, and PTEC8; Figure S13: FT-IR spectra of compounds and the blend systems; Figure S14: Photoluminescent emission spectra of T5E36/PTEC5, T5E36/PTEC6, T5E36/PTEC7, and T5E36/PTEC8; Figure S15: Double logarithmic plots of transient current (I) versus time (t) at 20 °C and an applied electric field of E = 2 × 10^4^ V/cm for T5E36/PTEC6, T5E36/PTEC7, and T5E36/PTEC8; Table S2: UV absorption edge wavelength and optical bandgap of the compounds and the blend systems. Figure S16: (a) HOMO and (b) LUMO frontier molecular orbitals of compound T5E36. Figure S17: (a) HOMO and (b) LUMO frontier molecular orbitals of compound PTEC5. Figure S18: (a) HOMO and (b) LUMO frontier molecular orbitals of compound PTEC6. Figure S19: (a) HOMO and (b) LUMO frontier molecular orbitals of compound PTEC7. Figure S20: (a) HOMO and (b) LUMO frontier molecular orbitals of compound PTEC8.
