# Supplementary material for: Construction of Donor–Acceptor Heterojunctions via Microphase Separation of Discotic Liquid Crystals with Ambipolar Transport
Source: Molecules. 2025 Aug 21;30(16):3441. doi: 10.3390/molecules30163441 (PMC12388344; doi:10.3390/molecules30163441)
Supplement: Supplementary file 1 [file molecules-30-03441-s001.zip › molecules-3719510-supplementary.pdf]

Supporting information for

Construction of Donor–Acceptor Heterojunctions via Microphase Separation of Discotic Liquid Crystals with Ambipolar Transport

Heng Liu[1,†], Ming Si Xie[1,†], Yao Hong Liu[1], Gao Jun Jia[1], Rui Juan Liao[1], Ao Zhang[1], Yi Fang[1], Xiao Li Song[1], Chun Xiu Zhang[1,*], and Hai Feng Yu[2,*].

[1] School of Printing and Packaging Engineering, Beijing Institute of Graphic Communication, Beijing 102600, China.
[2] Key Laboratory of Polymer Chemistry and Physics of Ministry of Education, School of Materials Science and Engineering, Peking University, Beijing 100871, China
* Corresponding authors
* Correspondence: Prof. Dr. C. Zhang: zhangchunxiu@bigc.edu.cn; Prof. Dr. H. Yu: yuhaifeng@pku.edu.cn
† These authors contributed equally to this work

Section S1. Materials and Methods……1

Section S2. Synthesis and characterization……1

Section S3. Reconstruction of Relative Electron Density Map……4

Figure S1. ^{1}H-NMR spectrum of T5E36……5

Figure S2. ^{1}H-NMR spectrum of PTEC5……5

Figure S3. ^{1}H-NMR spectrum of PTEC6……6

Figure S4. ^{1}H-NMR spectrum of PTEC7……6

Figure S5. ^{1}H-NMR spectrum of PTEC8……7

Figure S6. ^{13}C-NMR spectrum of T5E36……7

Figure S7. ^{13}C-NMR spectrum of PTEC5……8

Figure S8. ^{13}C-NMR spectrum of PTEC6……8

Figure S9. ^{13}C-NMR spectrum of PTEC7……9

Figure S10.^{13}C-NMR spectrum of PTEC8……9

Figure S11.DSC thermogram of T5E36 recorded during heating and cooling cycles at a rate of 10°C/min……10

Figure S12. WAXD patterns of PTEC5 at 30 °C, PTEC6 at 30 °C, PTEC7at 25 °C and PTEC8at 30 °……10

Table S1. Summary and detailed indexation of the complementary WAXD data for T5E36, PTEC5, PTEC6, PTEC7, PTEC8……11

Figure S13.FT-IR spectra of compounds and the blend system……12

Figure S14.Photoluminescent emission spectra of T5E36/PTEC5, T5E36/PTEC6, T5E36/PTEC7, and T5E36/PTEC8..12

Figure S15.Double logarithmic plots of transient current (I) versus time (t) at 20 °C and an applied electric field of E = 2 × 10^4 V/cm for T5E36/PTEC6, T5E36/PTEC7, and T5E36/PTEC8...........................12

Table S2. UV absorption edge wavelength and optical bandgap of the compounds and the blend systems..13

Figure S16. (a) HOMO and (b) LUMO frontier molecular orbitals of compound T5E36.........................13

Figure S17. (a) HOMO and (b) LUMO frontier molecular orbitals of compound PTEC5........................13

Figure S18. (a) HOMO and (b) LUMO frontier molecular orbitals of compound PTEC6........................14

Figure S19. (a) HOMO and (b) LUMO frontier molecular orbitals of compound PTEC7........................14

Figure S20. (a) HOMO and (b) LUMO frontier molecular orbitals of compound PTEC8........................14

Section S1: Materials and Methods

All chemicals were purchased from Aladdin (Shanghai, China), and all solvents were obtained from Sigma-Aldrich (Saint Louis, MO, USA). Unless otherwise specified, all chemicals and solvents were used without further purification. The products were purified by column chromatography using silica gel 60 (200 - 300 mesh, ASTM standard) from Aladdin (Shanghai, China), and the reaction progress was monitored by thin-layer chromatography (TLC) on silica gel 60 F254 glass plates from Aladdin (Shanghai, China). ^{1}HNMR spectra were recorded on a Bruker DMX 400 MHz spectrometer (Bruker Corporation, Billerica, MA, USA) using $CDCl_3$ as the solvent. Chemical shifts (δ) are reported in parts per million (ppm) relative to tetramethylsilane (TMS) as the internal standard. Signal multiplicities are designated as follows: s = singlet, d = doublet, t = triplet, and m = multiplet. The ^{13}C-NMR spectra of PTEC5, PTEC6, and PTEC7 were recorded in $CDCl_3$ using a Bruker AVANCE 400 MHz NMR spectrometer (Bruker Corporation, Billerica, MA, USA), while the spectrum of PTEC8 was obtained using a Bruker AVANCE 500 MHz NMR spectrometer (Bruker Corporation, Billerica, MA, USA). Samples for FT-IR analysis were prepared using KBr pellets, and spectra were recorded on a Shimadzu FTIR-8400 spectrometer (Shimadzu Corporation, Kyoto, Japan). Differential scanning calorimetry (DSC) measurements were conducted on a Netzsch 200 F3 differential scanning calorimetry (Netzsch Analyzing & Testing GmbH, Wittelsbacherstraße, Germany). Approximately 5 mg of each sample was sealed in an aluminum crucible and scanned under a nitrogen atmosphere at a heating rate of 10 °C min^{-1} to assess thermal properties. Polarized optical microscopy (POM) was carried out using a Leica DM4500 P microscope (Leica Microsystems GmbH, Wetzlar, Germany) equipped with a Linkam TMS 94 hot stage (Linkam Scientific Instruments Ltd, UK)) to observe the optical textures, upon cooling at a rate of 10 °C/min. Wide-angle X-ray diffraction (WAXD) measurements were performed on a Bruker D8 Advance diffractometer (Bruker Corporation, Billerica, MA, USA) equipped with a temperature controller at a scanning rate of 10 °C min^{-1}. Powder samples were placed on aluminum foil, and the resulting XRD patterns were analyzed using appropriate software. UV–Vis absorption spectra were recorded using a Agilent Cary 300 UV−Vis spectrophotometer (Agilent Technologies, Santa Clara, CA, USA) over the wavelength range of 0-800nm. Solution samples were prepared by dissolving the compounds in DCM and placed in a quartz cuvette. Baseline correction was performed using the corresponding solvent as reference. All measurements were conducted at room temperature under ambient conditions. Photoluminescence (PL) spectra were recorded using a Hitachi F-4700 fluorescence spectrophotometer (Hitachi High-Technologies Corporation, Tokyo, Japan) equipped with a xenon lamp as the excitation source. The samples were prepared by dissolving the compounds in dichloromethane (DCM). The measurements were carried out in quartz cuvette at room temperature under ambient conditions.

Section S2: Synthesis and Characterizations

Synthesis of 3,6-di(ethoxycarbonyl)-2,7,10,11-tetrapentyloxytriphenylene(T5E36): Under a nitrogen atmosphere, 3,6-dihydroxy-2,7,10,11-tetrapentyloxyphenanthrene (0.5 g), triethylamine (1.2 mL), and acetyl chloride (2 mL) were dissolved in dry dichloromethane and refluxed at 40 °C for 24 h. The reaction

was monitored by thin-layer chromatography (TLC) using ethyl acetate/petroleum ether (1:4) as the eluent. After completion, the reaction mixture was purified by silica gel column chromatography, employing ethyl acetate/dichloromethane/petroleum ether (1:2:24) as the eluent. The purified product was recrystallized from ethanol, yielding the desired compound as a solid with a yield of 95%. ^{1}H NMR (400 MHz, Chloroform-d) δ 7.99 (s, 2H), 7.81 (d, J = 2.2 Hz, 4H), 4.22 (dt, J = 12.7, 6.5 Hz, 8H), 2.38 (s, 6H), 2.04 – 1.84 (m, 8H), 1.60 – 1.41 (m, 16H), 0.98 (td, J = 7.2, 3.6 Hz, 12H). ^{13}C NMR (101 MHz, Chloroform-d)δ 169.16, 149.70, 149.60, 139.99, 127.85, 124.05, 122.93, 116.91, 107.56, 106.11, 77.22, 69.65, 68.92, 29.09, 28.95, 28.38, 28.23, 22.58, 22.47, 20.65, 14.12, 14.09

Synthesis of perylene-3,4,9,10-tetracarboxylic acid potassium salt：3,4,9,10-Tetrahydroxyanthracene (7.84 g, 20 mmol) was added to a 250 mL three-necked flask, followed by the addition of deionized water (100 mL). The mixture was stirred magnetically for 10 minutes. Subsequently, sodium hydroxide (6 g, 106 mmol) was added, and the mixture was heated under reflux for 4 hours until the red solid completely dissolved. Upon cooling to room temperature, the reaction mixture was slowly poured into an acidified water bath. A yellow solid precipitated and was collected by filtration. After drying, 11.5 g of product was obtained, corresponding to a 99% yield.

Synthesis of 3,4,9,10-tetrakis(n-pentyl ester) perylene tetracarboxylate (PTEC5): In a three-necked round-bottom flask, potassium perylene-3,4,9,10-tetracarboxylate (10.0 g, 17.24 mmol), potassium carbonate (20.0 g, 144.7 mmol), methyltrioctylammonium chloride (2.1 g, 5.187 mmol), potassium iodide (5.187 mmol), and deionized water (140 mL) were added sequentially. The mixture was stirred magnetically for 10 minutes, after which n-pentyl bromide (20.80 g, 137.76 mmol) was added dropwise via a constant-pressure dropping funnel. The reaction mixture was refluxed for 24 hours, then allowed to cool to room temperature. The resulting mixture was extracted three times with dichloromethane (CH_2Cl_2). The combined organic layers were dried over anhydrous sodium sulfate overnight. After removal of the solvent under reduced pressure, the crude product was purified by column chromatography using CH_2Cl_2 as the eluent, followed by recrystallization from ethanol. The product was obtained as a golden solid (10.1 g), corresponding to a yield of 89%. The final compound was dried in an oven for 24 hours prior to characterization. ^{1}H NMR (400 MHz, Chloroform-d) δ 8.36 (d, *J* = 8.0 Hz, 4H), 8.08 (d, *J* = 7.9 Hz, 4H), 4.32 (t, *J* = 6.9 Hz, 8H), 1.79 (p, *J* = 7.0 Hz, 8H), 1.46 – 1.34 (m, 16H), 1.00 – 0.90 (m, 12H). ^{13}C NMR (101 MHz, Chloroform-d) δ 168.53, 132.99, 130.45, 130.38, 128.96, 128.78, 121.36, 65.63, 28.32, 28.17, 22.42, 14.01.

Synthesis of 3,4,9,10-Tetrakis(n-hexyl ester) perylene tetracarboxylate (PTEC6): Under magnetic stirring, potassium perylene-3,4,9,10-tetracarboxylate (10.0 g, 17.24 mmol), trioctylmethylammonium chloride (2.1 g, 5.187 mmol), potassium iodide (0.57 g, 5.178 mmol), and deionized water (140 mL) were sequentially added to a 250 mL three-necked flask equipped with a spherical condenser and a constant-pressure dropping funnel. The mixture was stirred magnetically for 20 minutes until the solids were fully dissolved. Monobromohexane (22.74 g, 137.76 mmol) was then added dropwise via the dropping funnel. The reaction mixture was refluxed at 100 °C for 48 hours. Reaction progress was monitored by observing the precipitation of an orange-red solid when aliquots were added dropwise into water; increased

precipitation indicated a more complete reaction. Upon completion, the mixture was cooled to room temperature. The resulting solid was collected by filtration using a Büchner funnel to afford an orange-red crude product. The crude material was purified by column chromatography using dichloromethane (CH_2Cl_2) as the eluent. The solvent was removed by rotary evaporation, and the product was recrystallized from ethanol. The resulting solid was filtered again through a Büchner funnel, and the filter cake was rinsed with ethanol to yield 11.2 g of a golden-yellow filamentous solid. The product was dried in an oven for 24 hours, affording a final yield of 88%. ^{1}H NMR (400 MHz, Chloroform-*d*) δ 8.35 (d, *J* = 8.0 Hz, 4H), 8.07 (d, *J* = 7.9 Hz, 4H), 4.32 (t, *J* = 6.9 Hz, 8H), 1.83 – 1.75 (m, 8H), 1.49 – 1.32 (m, 24H), 0.94 – 0.86 (m, 12H). ^{13}C NMR (101 MHz, Chloroform-d) δ 168.53, 133.07, 130.50, 130.42, 129.03, 128.84, 121.39, 65.66, 31.53, 28.57, 25.69, 22.57, 14.03.

Synthesis of 3,4,9,10-Tetrakis(n-heptyl ester) perylene tetracarboxylate (PTEC7): Under magnetic stirring, potassium perylene-3,4,9,10-tetracarboxylate (10.0 g, 17.24 mmol), trioctylmethylammonium chloride (2.1 g, 5.187 mmol), potassium iodide (0.57 g, 5.178 mmol), and deionized water (140 mL) were sequentially added to a 250 mL three-necked flask equipped with a spherical condenser and a constant-pressure dropping funnel. The mixture was stirred magnetically for 20 minutes until the solids were fully dissolved. Heptane bromide (24.68 g, 137.76 mmol) was then added dropwise via the dropping funnel. The reaction mixture was refluxed at 100 °C for 48 hours. Progress of the reaction was monitored by withdrawing small aliquots and adding them dropwise to water; the formation of an orange-red precipitate was indicative of product formation and reaction completion. After the reaction was complete, the mixture was cooled to room temperature. The resulting orange-red solid was collected by filtration using a Büchner funnel. The crude product was purified by column chromatography using dichloromethane (CH_2Cl_2) as the eluent. The solvent was removed under reduced pressure via rotary evaporation, and the product was recrystallized from ethanol. The resulting solid was filtered again through a Büchner funnel, and the filter cake was rinsed with ethanol to afford 12.25 g of a golden-yellow filamentous solid. The product was dried in an oven for 24 hours, affording a final yield of 91%. ^{1}H NMR (400 MHz, Chloroform-*d*) δ 8.35 (d, *J* = 8.0 Hz, 4H), 8.07 (d, *J* = 7.9 Hz, 4H), 4.31 (t, *J* = 6.9 Hz, 9H), 1.78 (p, *J* = 7.0 Hz, 8H), 1.50 – 1.26 (m, 32H), 0.93 – 0.85 (m, 12H). ^{13}C NMR (101 MHz, Chloroform-d) δ 168.53, 132.99, 130.45, 130.39, 128.97, 128.78, 121.36, 65.65, 31.76, 29.03, 28.63, 26.00, 22.62, 14.08.

Synthesis of 3,4,9,10-Tetrakis(n-octyl ester) perylene tetracarboxylate (PTEC8): To a 250 mL three-necked flask equipped with a magnetic stirrer, constant-pressure dropping funnel, and spherical condenser, potassium perylene-3,4,9,10-tetracarboxylate (10.0 g, 17.24 mmol), trioctylmethylammonium chloride (2.1 g, 5.187 mmol), potassium iodide (0.57 g, 5.178 mmol), and deionized water (140 mL) were added sequentially. The mixture was stirred magnetically for 20 minutes until complete dissolution of the solid. Octane bromide (26.60 g, 137.76 mmol) was then added dropwise via the dropping funnel, and the reaction mixture was refluxed at 100 °C for 48 hours. Reaction progress was monitored by withdrawing aliquots and adding them dropwise into water; the formation of an orange-red precipitate indicated ongoing product formation. A higher amount of precipitate suggested greater reaction completion. Upon

completion, the mixture was cooled to room temperature. The precipitated orange-red solid was collected by filtration through a Büchner funnel. The crude product was purified by column chromatography using dichloromethane (CH_2Cl_2) as the eluent. After removal of the solvent under reduced pressure by rotary evaporation, the purified compound was recrystallized from ethanol. The product was then filtered again through a Büchner funnel and the filter cake was rinsed with ethanol to afford 13.65 g of a golden-yellow filamentous solid. The final product was dried in an oven for 24 hours, yielding 90%. ^{1}H NMR (400 MHz, Chloroform-*d*) δ 8.32 (d, *J* = 8.0 Hz, 4H), 8.06 (d, *J* = 7.9 Hz, 4H), 4.32 (t, *J* = 6.9 Hz, 8H), 1.78 (p, *J* = 7.0 Hz, 8H), 1.48 – 1.20 (m, 42H), 0.99 – 0.81 (m, 13H). ^{13}C NMR (126 MHz, Chloroform-d) δ 168.52, 133.01, 130.46, 130.40, 128.99, 128.79, 121.36, 65.66, 31.82, 29.33, 29.22, 28.62, 26.04, 22.66, 14.11.

Section S3. **Reconstruction of Relative Electron Density Map**

On the basis of XRD data, the reconstruction of relative electron density distribution in real space is carried out using the formula for 2D Fourier transformation:

$$\varrho(x, y) - \varrho 0 = \sum_{hk} F(hk) \exp[-2\pi(hx + ky)]$$

where ϱ0 is the average electron density and x, y are the fractional coordinates of a point in the unit cell. F(*hk*) is the complex structure factor and its modulus is related to the diffraction intensities I(*hk*) by $|F(hk)| = \sqrt{I(hk)}$. Note that diffraction intensities need to be multiplicity corrected. Also the summation is executed over all possible integer combination of (*hk*) except for (00). If the lattice is chosen as centrosymmetric, the structure factor becomes real and is given by F(*hk*)=±|F(*hk*)|, with corresponding possible phase of 0 (+) or π (–). Then the electron density can be expressed as:

$$\varrho(x, y) - \varrho 0 = \sum_{hk} \pm \sqrt{I(hk)} \cos[-2\pi(hx + ky)]$$

Considering the hexagonal columnar phase, three clear peaks can be assigned for T5E36/PTEC6, T5E36/PTEC7 and T5E36/PTEC8. These peaks are related to diffraction (10), (11), (20). For the c2mm rectangular columnar phase of T5E36/PTEC5, its peaks are related to the diffraction patterns (20), (10), (22). The electron density maps have been calculated using all possible phase combinations for the diffractions.

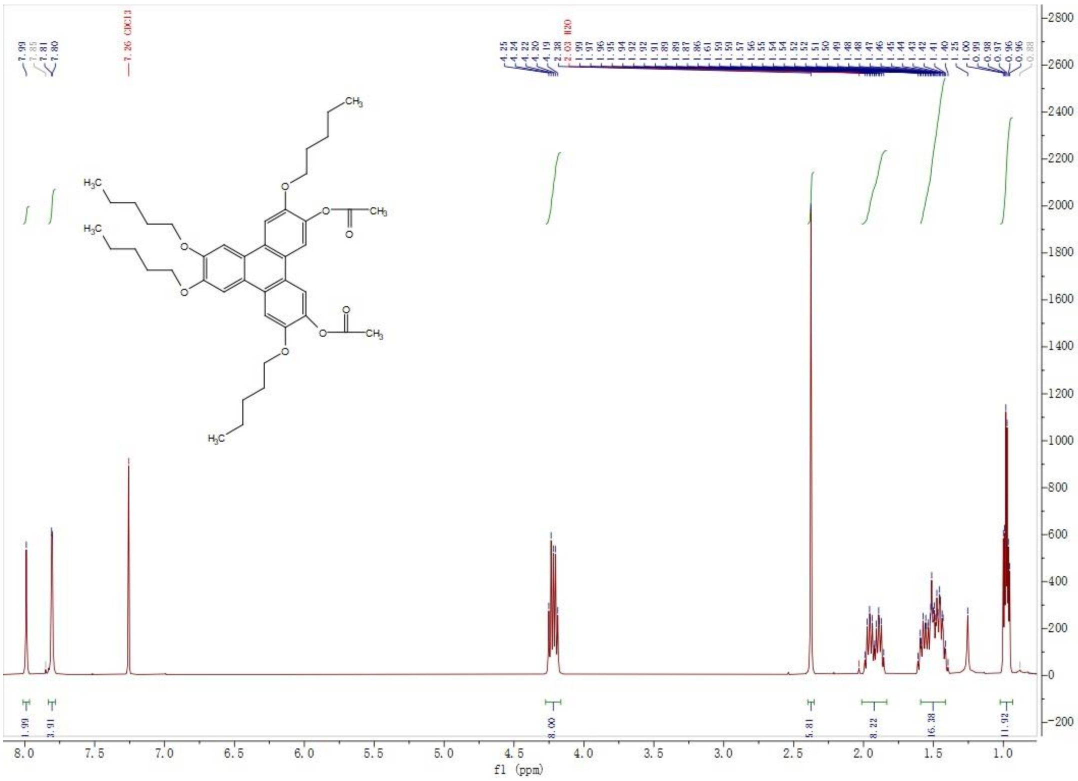

Figure S1. ¹H-NMR spectrum of T5E36. Note: The signal observed at approximately 1.25 ppm in the ¹H NMR spectrum is attributed to residual HDO.

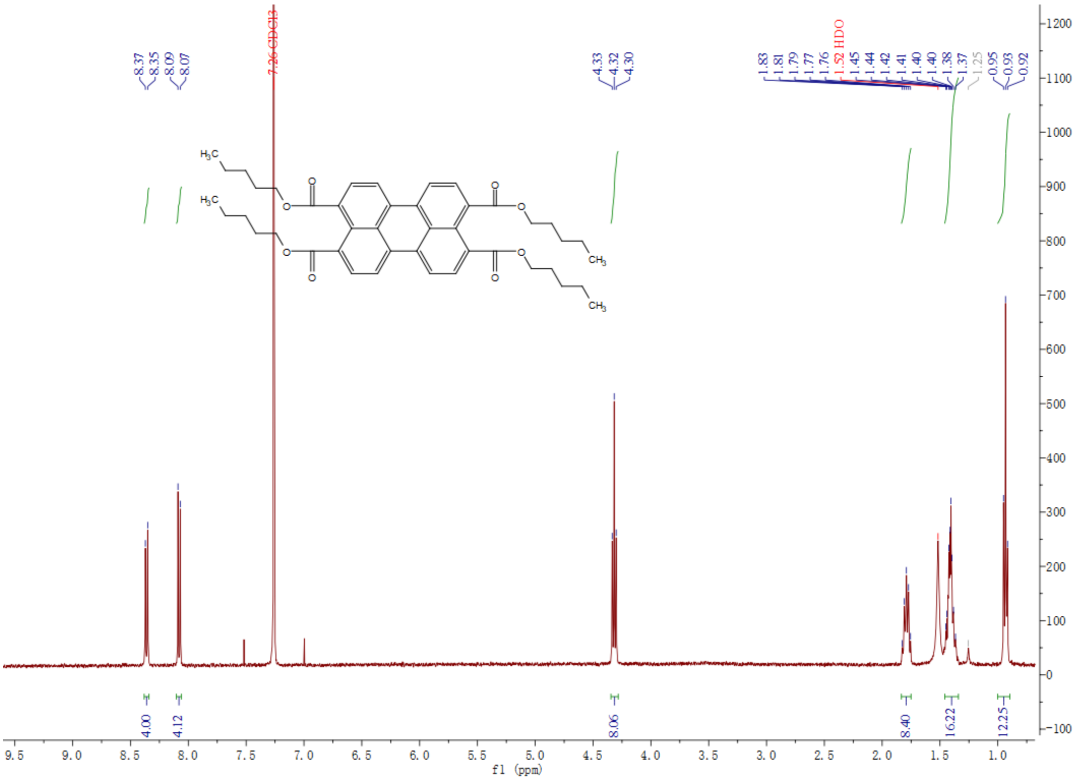

Figure S2. ¹H-NMR spectrum of PTEC5.

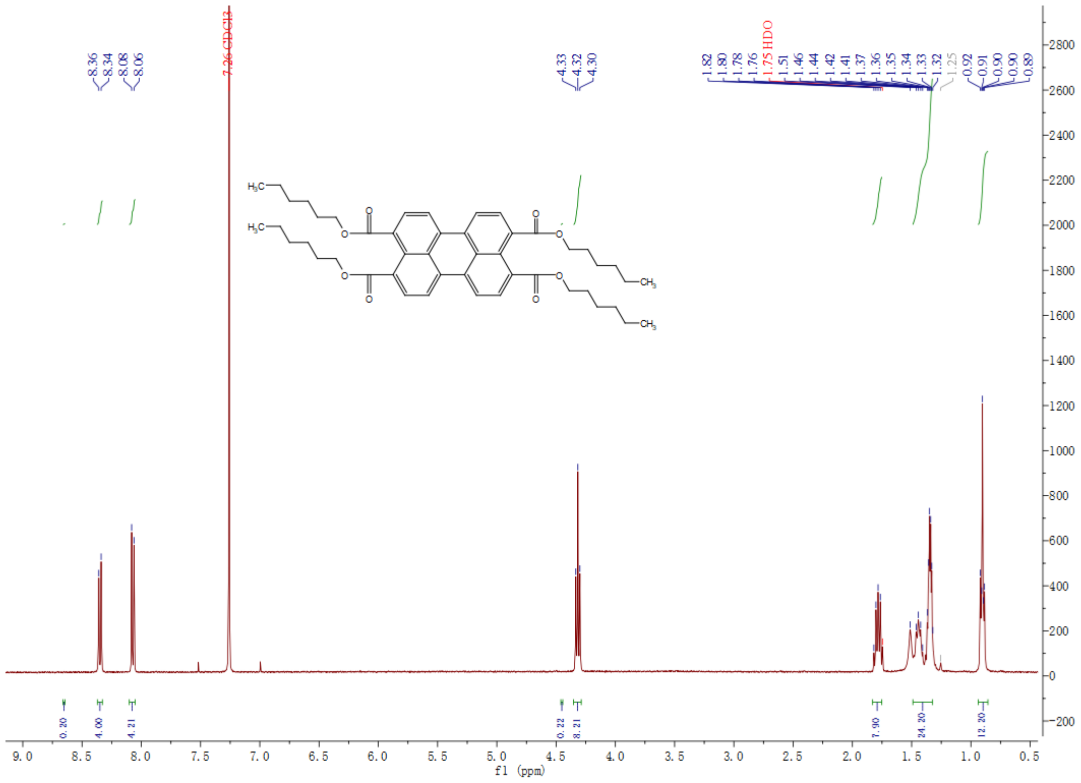

Figure S3. ^{1}H-NMR spectrum of PTEC6.

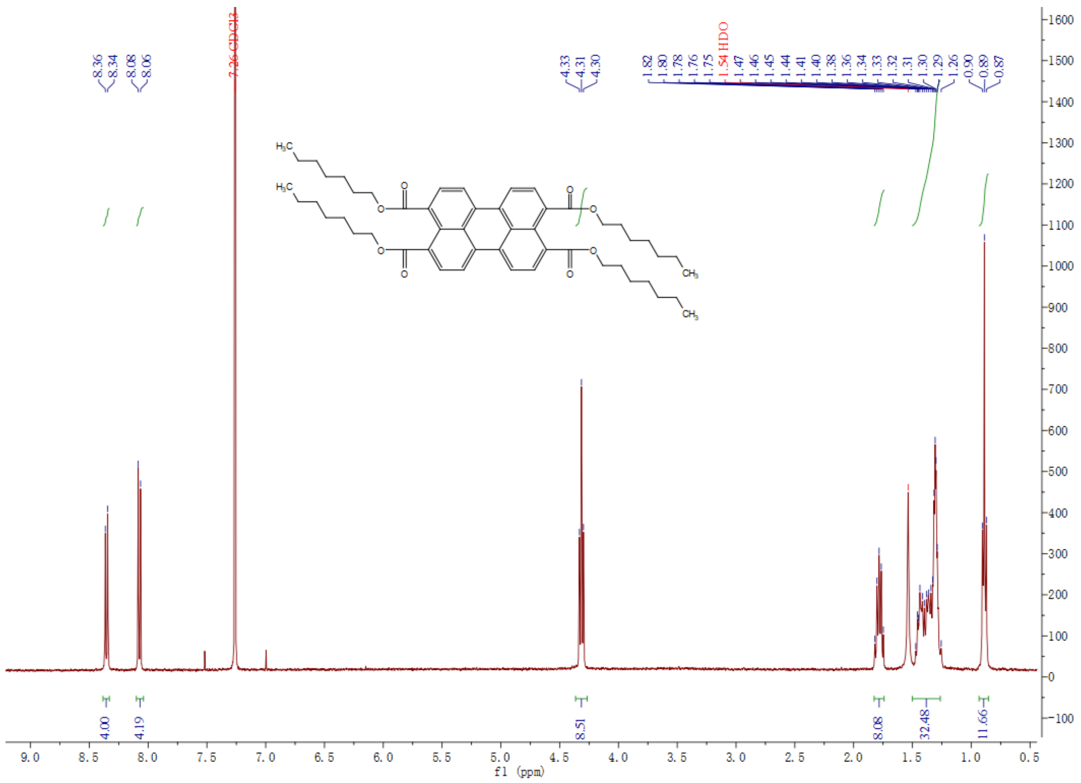

Figure S4. ^{1}H-NMR spectrum of PTEC7.

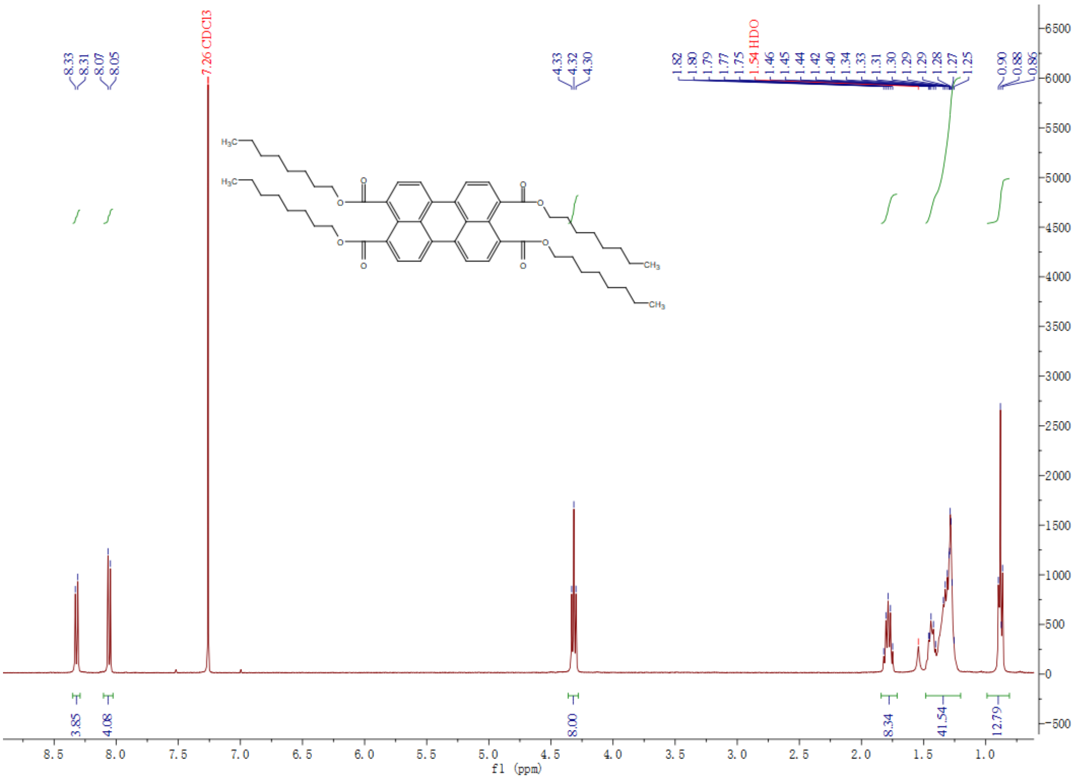

Figure S5. ^{1}H-NMR spectrum of PTEC8.

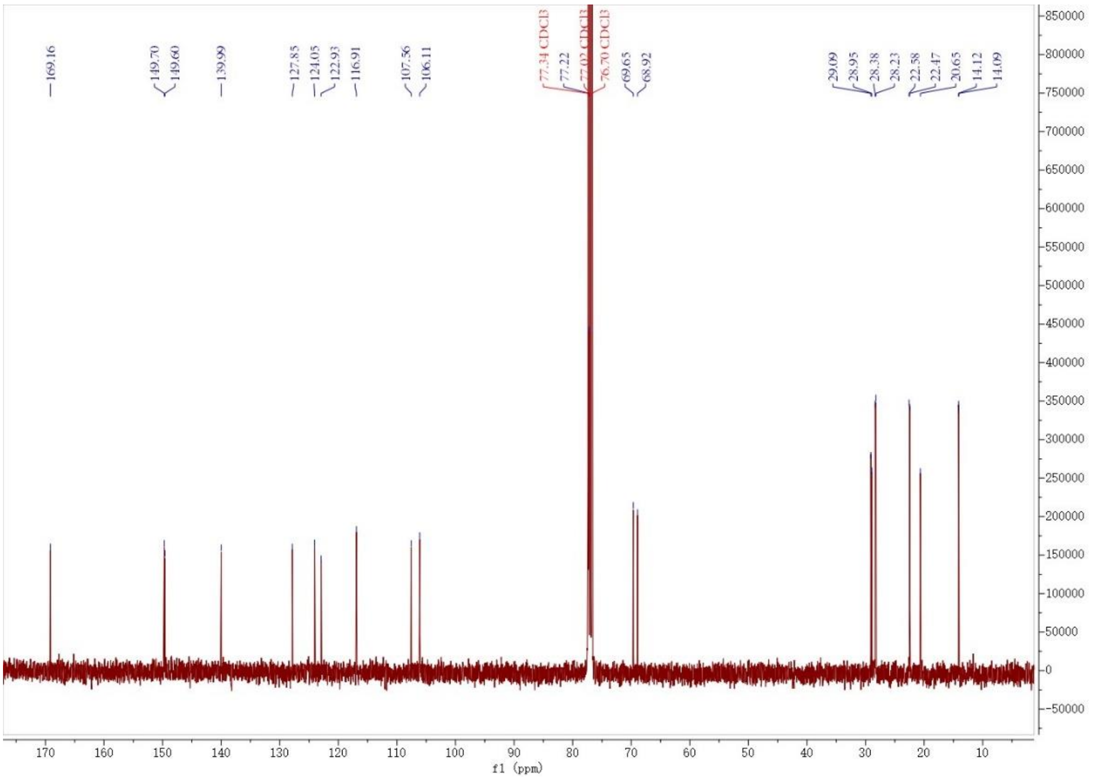

Figure S6. ^{13}C-NMR spectrum of T5E36

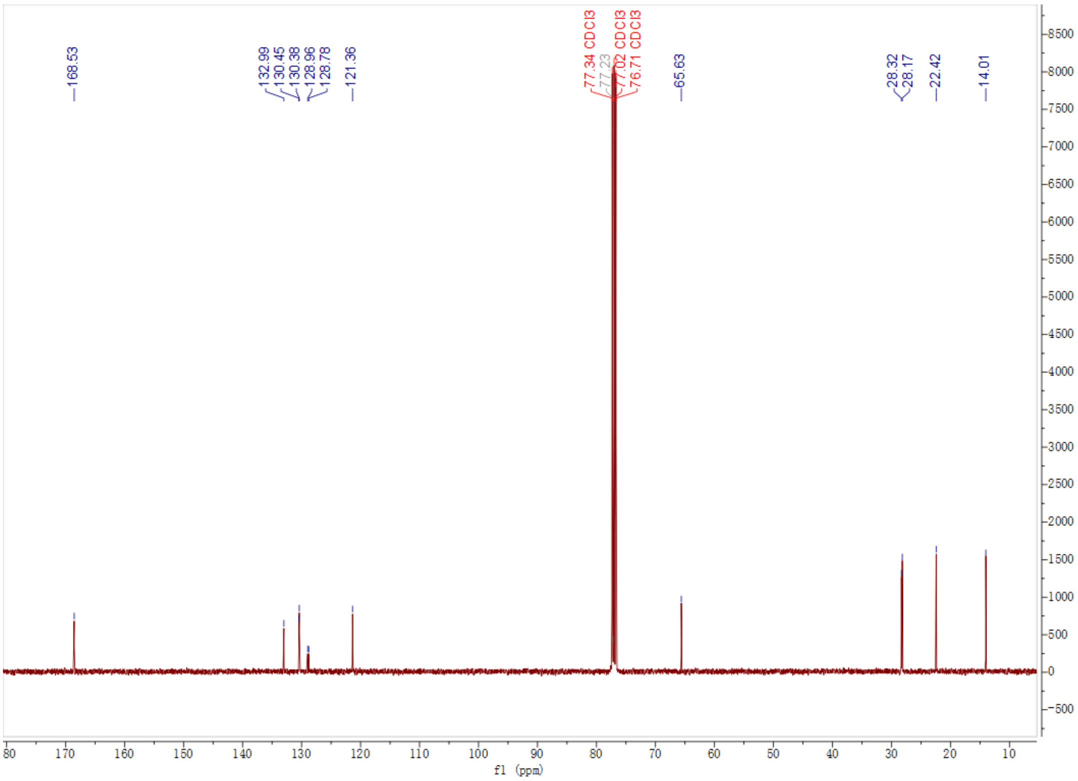

Figure S7. ^{13}C-NMR spectrum of PTEC5

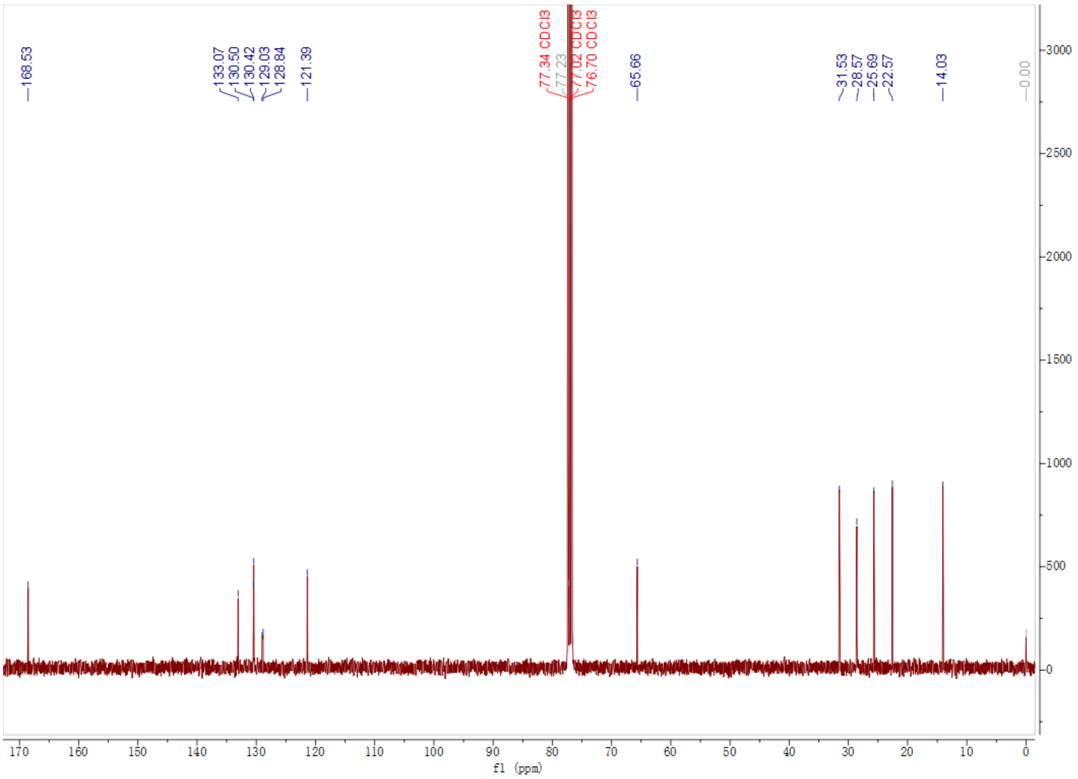

Figure S8. ^{13}C-NMR spectrum of PTEC6

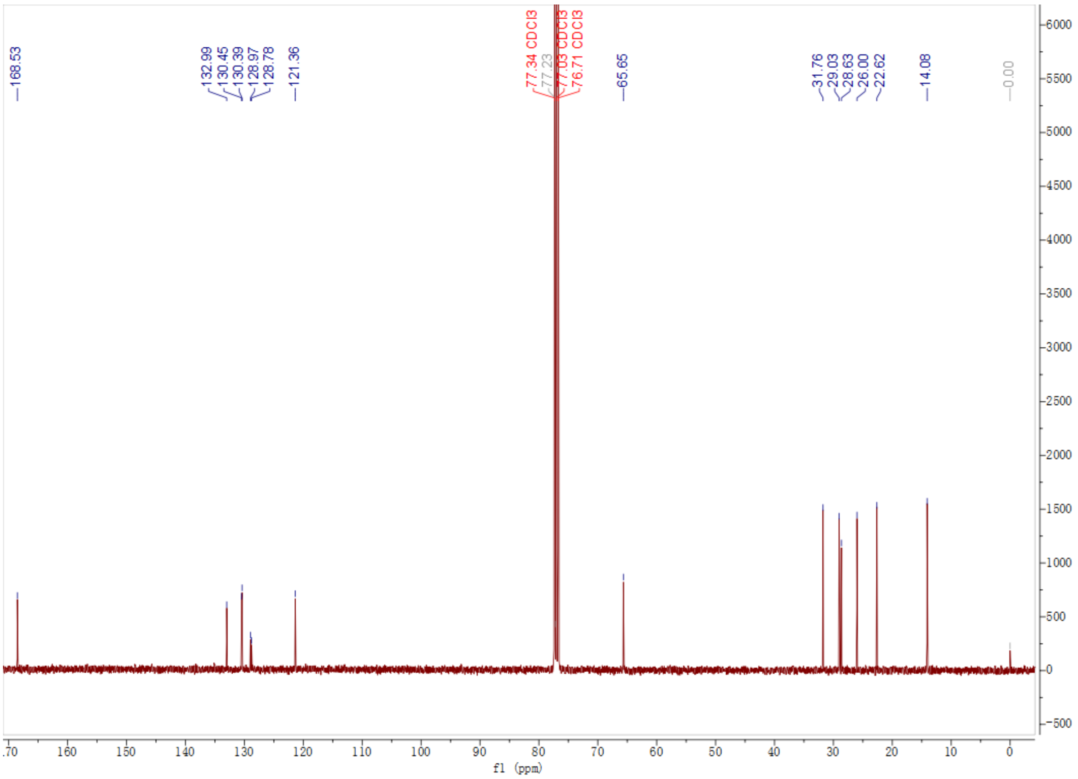

Figure S9. ^{13}C-NMR spectrum of PTEC7.

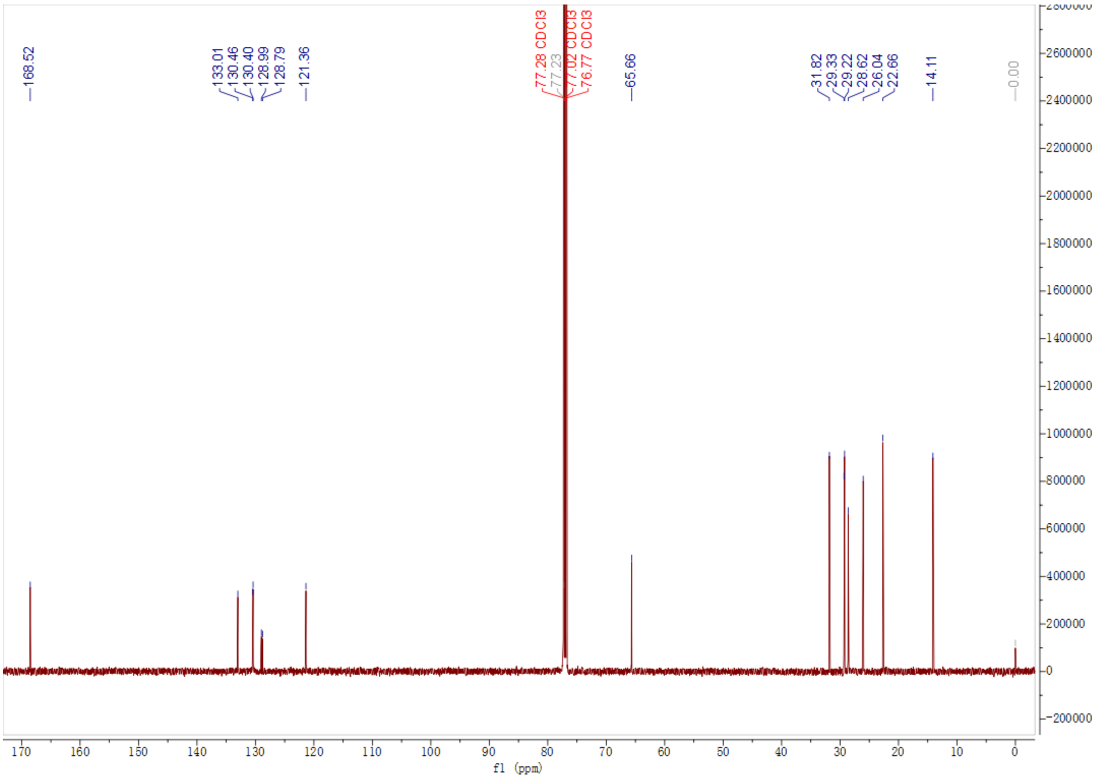

Figure S10. ^{13}C-NMR spectrum of PTEC8.

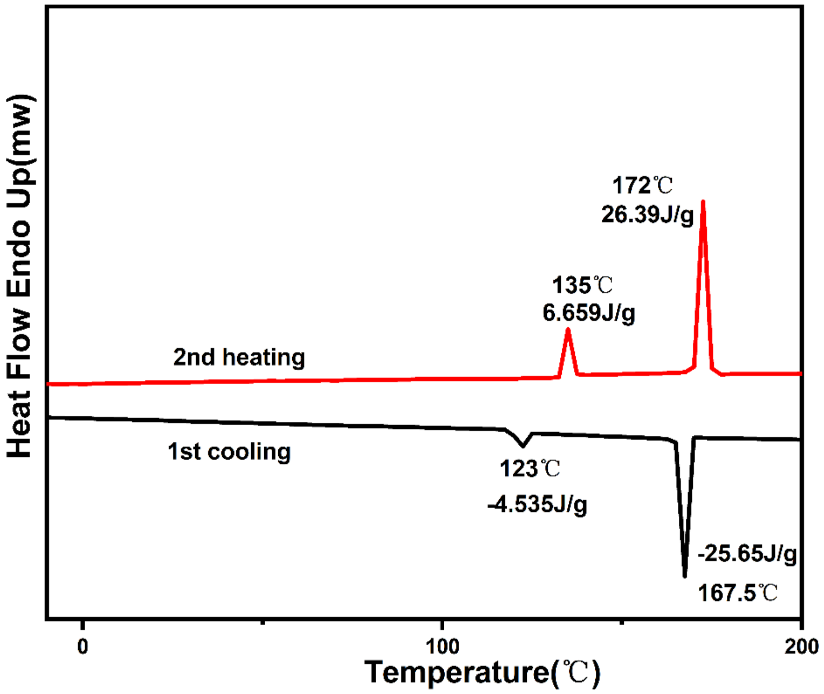

Figure S11. DSC thermogram of T5E36 recorded during heating and cooling cycles at a rate of 10°C/min.

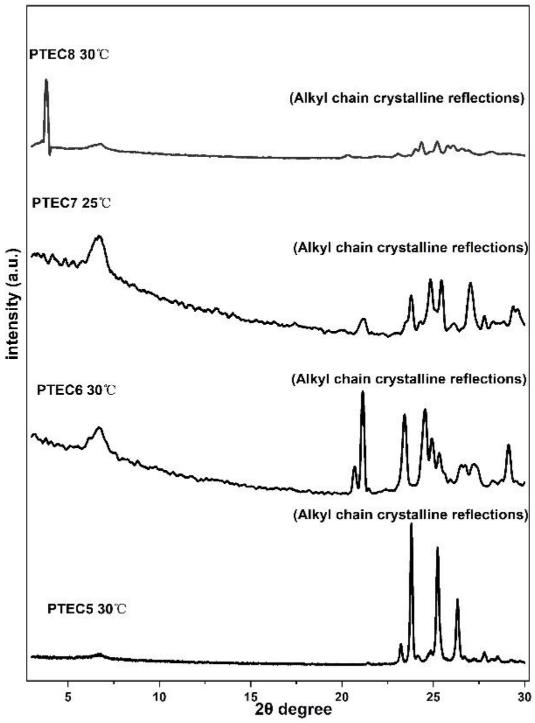

Figure S12. WAXD patterns of PTEC5 at 30 °C,PTEC6 at 30 °C, PTEC7at 25 °C and PTEC8at 30 °C. As observed from the WAXD patterns, PTEC5, PTEC6, PTEC7, and PTEC8 all exhibit multiple sharp and intense diffraction peaks in the 2θ range of 20°–30°, corresponding to d-spacings of approximately 3.0–4.4 Å. Such high-angle crystalline diffraction peaks are typically attributed to the crystallization of alkyl chains, indicating that these samples exhibit pronounced crystalline characteristics at lower temperatures.

Table S1. Summary and detailed indexation of the complementary WAXD data for T5E36，PTEC5, PTEC6, PTEC7, PTEC8.

Sample	d-Spacing (Å)	Miller Index (*hkl*)	Phase (lattice constants)
T5E36 (40°C)	16.36	（100）	Col_h（a=18.89 Å）
Col_h/*p*6*mm*	8.24	（200）	
	6.39	（210）	
	5.46	（300）	
	4.67	（310）	
	4.23	（*h*c）	
	3.67	（hπ）	
T5E36(125°C)	16.56	（100）	Col_h（a=19.13 Å）
Col_h/*p*6*mm*	9.53	（110）	
	8.34	（200）	
	6.27	（210）	
	3.42	（*h*π）	
T5E36(160°C)	16.61	（100）	Col_h（a=19.18Å）
Col_h/*p*6*mm*	6.33	(210)	
	5.58	(300)	
	3.52	(*h*π)	
PTEC5(130°C)	16.08	（100）	Col_h（a=18.56Å）
Col_h/*p*6*mm*	9.68	（110）	
	8.02	（200）	
	6.01	（210）	
PTEC6(90°C)	16.91	（100）	Col_h（a=19.52 Å）
Col_h/*p*6*mm*	9.78	（110）	
	8.44	（200）	
	6.41	（210）	
PTEC7(90°C)	18.20	（100）	Col_h（a=21.01Å）
Col_h/*p*6*mm*	10.44	（110）	
	9.16	（200）	
	6.88	（210）	
PTEC8(110°C)	18.90	（100）	Col_h（a=21.82Å）
Col_h/*p*6*mm*	10.88	（110）	
	9.37	（200）	
	7.19	（210）	

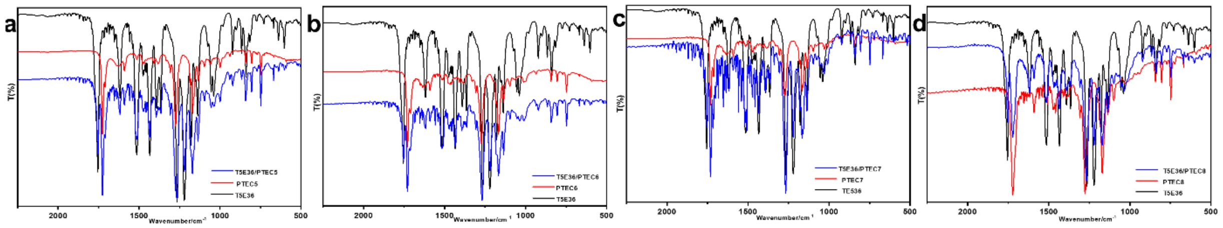

Figure S13. FT-IR spectra of the compounds and the blend systems: (a) T5E36/PTEC5, PTEC5, and T5E36; (b) T5E36/PTEC6, PTEC6 and T5E36; (c) T5E36/PTEC7, PTEC7, and T5E36; (d) T5E36/PTEC8, PTEC8, and T5E36.

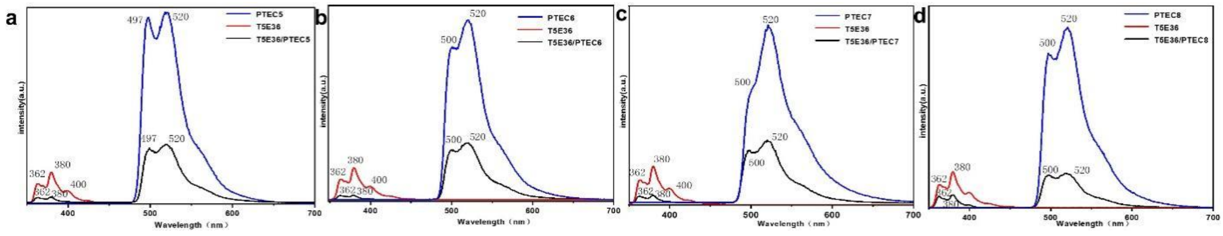

Figure S14. Photoluminescent emission spectra of (a) PTEC5, T5E36, T5E36/PTEC5, (b) PTEC6, T5E36, T5E36/PTEC6, (c) PTEC7, T5E36, T5E36/PTEC7, and (d) PTEC8, T5E36, T5E36/PTEC8. The excitation wavelength of T5E36 and the blend systems is 348 nm, and the excitation wavelength of PTECn is 493 nm.

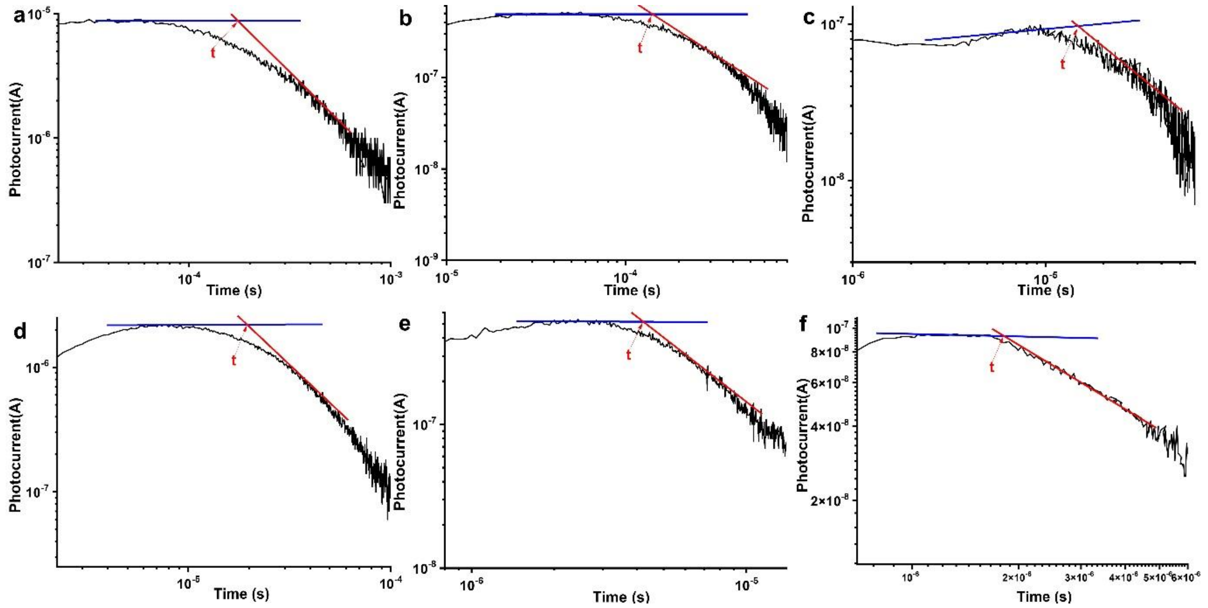

Figure S15. Double logarithmic plots of transient current (I) versus time (t) at 20 °C and an applied electric field of E = 2 × 10^4 V/cm for T5E36/PTEC6, T5E36/PTEC7, and T5E36/PTEC8: (a) hole mobility of T5E36/PTEC6, (b) electron mobility of T5E36/PTEC6, (c) hole mobility of T5E36/PTEC7, (d) electron mobility of T5E36/PTEC7, (e) hole mobility of T5E36/PTEC8, (f) electron mobility of T5E36/PTEC8

Table S2. The UV absorption edge wavelength and optical bandgap of the compounds and the blend systems

Compound	λ_{edge} (nm)	E_g(eV)
T5E36	400	3.1
PTEC5	521	2.38
PTEC6	525	2.36
PTEC7	536	2.31
PTEC8	534	2.32
T5E36/PTEC5	530	2.34
T5E36/PTEC6	542	2.29
T5E36/PTEC7	536	2.31
T5E36/PTEC8	534	2.32

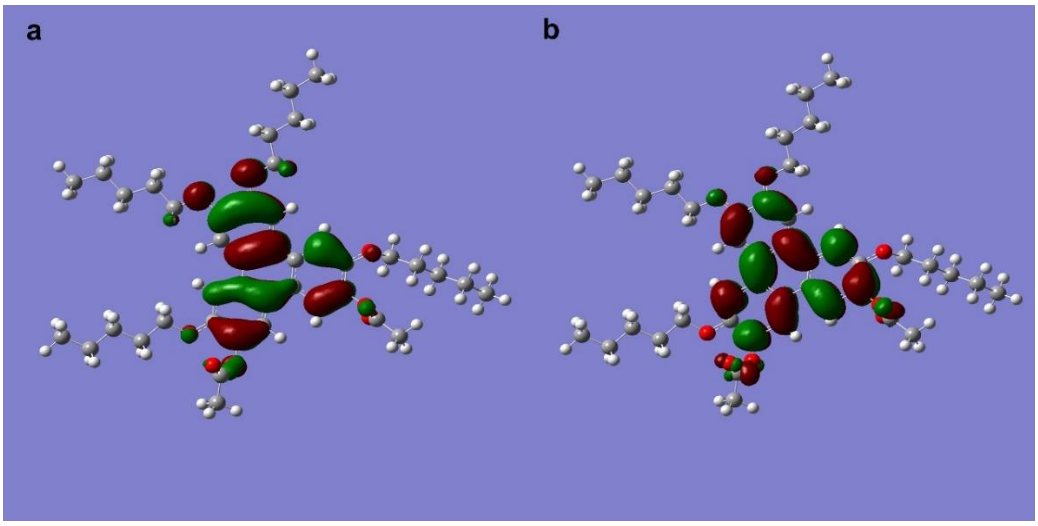

Figure S16. (a) HOMO and (b) LUMO frontier molecular orbitals of compound T5E36.

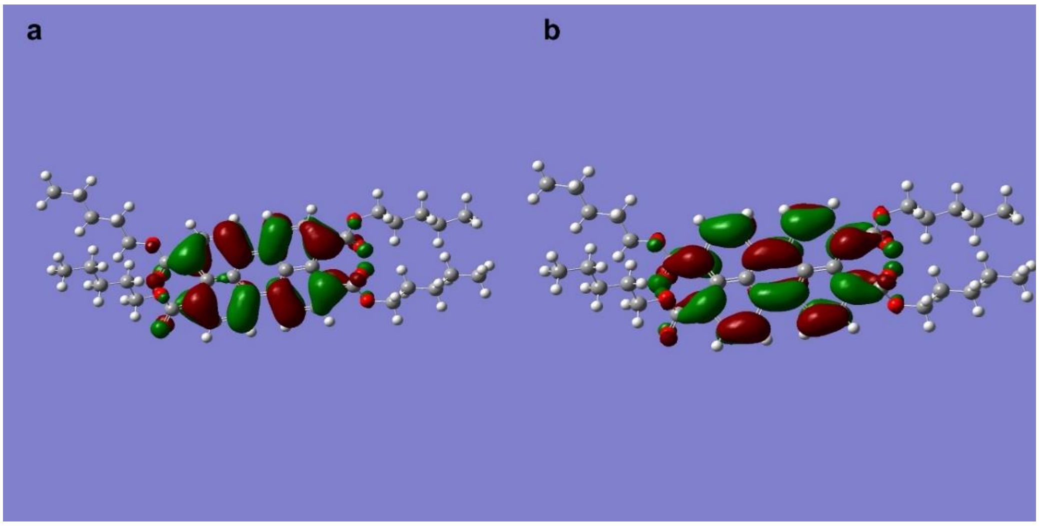

Figure S17. (a) HOMO and (b) LUMO frontier molecular orbitals of compound PTEC5.

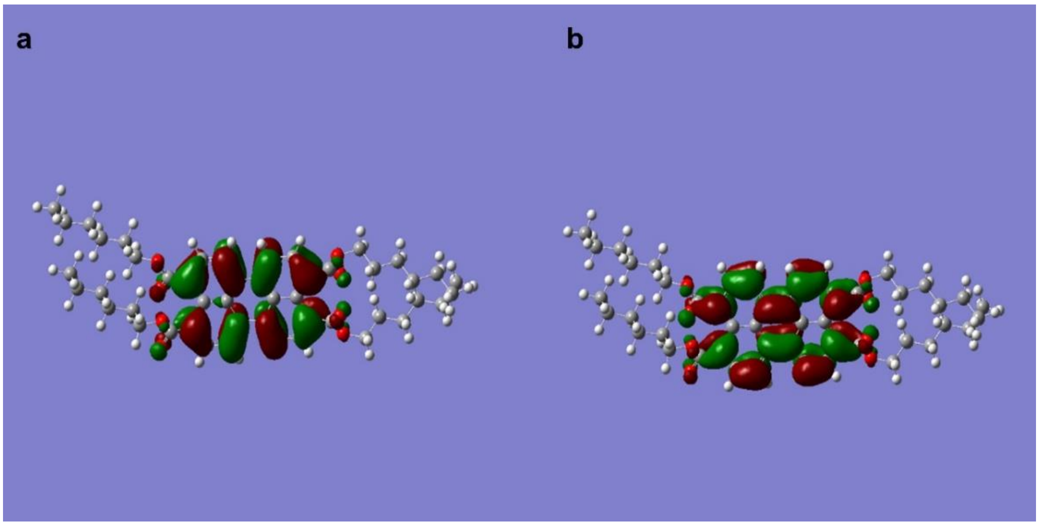

Figure S18. (a) HOMO and (b) LUMO frontier molecular orbitals of compound PTEC6.

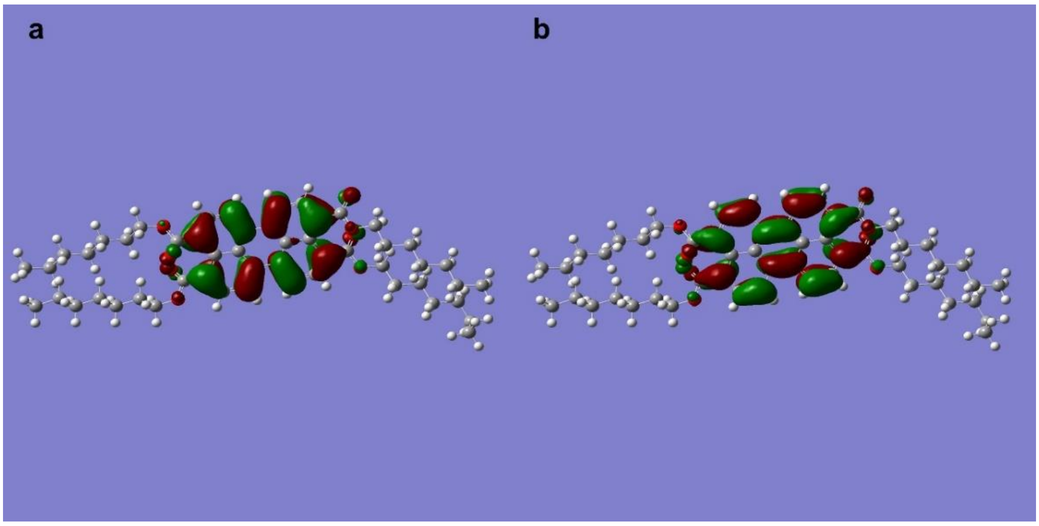

Figure S19. (a) HOMO and (b) LUMO frontier molecular orbitals of compound PTEC7.

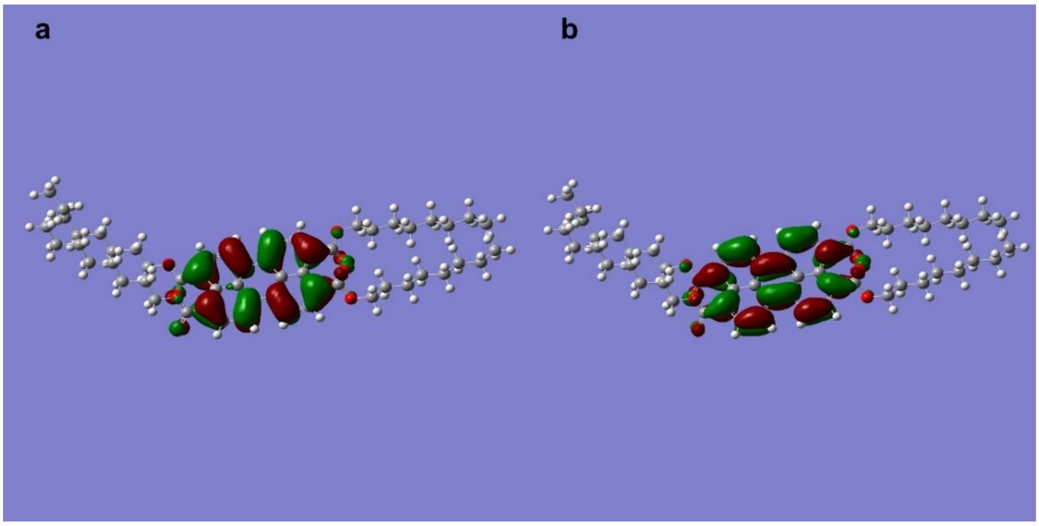

Figure S20. (a) HOMO and (b) LUMO frontier molecular orbitals of compound PTEC8.
